# Synthesis and Olfactory Properties of *Seco*-Analogues of Lilac Aldehydes

**DOI:** 10.3390/molecules26237086

**Published:** 2021-11-23

**Authors:** Vladimír Dacho, Peter Szolcsányi

**Affiliations:** Department of Organic Chemistry, Slovak University of Technology, Radlinského 9, SK-812 37 Bratislava, Slovakia; vladimirdacho@gmail.com

**Keywords:** terpenes, structural design, natural products, olfactory evaluation

## Abstract

Lilac aldehydes are considered as principal olfactory molecules of lilac flowers. We have designed, prepared, and evaluated a set of racemic *seco*-analogues of such natural products. The synthesis employs commercially available α-chloroketones as substrates that are transformed in four steps to target compounds. Their qualitative olfactory analysis revealed that the opening of the tetrahydrofuran ring leads to a vanishing of original flowery scent with the emergence of spicy aroma accompanied by green notes, and/or fruity aspects of novel *seco*-analogues. These results suggest the important osmophoric role of THF moiety for the generation of the typical flowery aroma associated with lilac aldehydes.

## 1. Introduction

**Figure 1:**
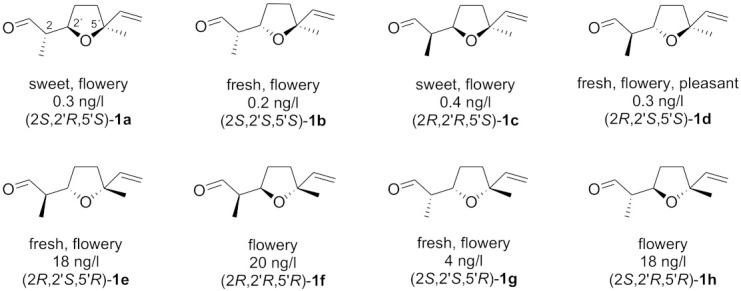
All possible stereoisomers of lilac aldehydes **1a**–**1h** with their respective odour thresholds.

Lilac aldehydes **1** (Figure 1) were first isolated in 1974 from an oil [1] of lilac flowers (*Syringa vulgaris* L., Oleaceae). Being considered as important phytovolatiles, structure elucidation and olfactometric analysis of all stereoisomers was performed [2]. Consequently, the biosynthesis of lilac aldehydes was proposed and investigated [3]. Since then, these naturally occurring monoterpenes were found in flowers of many other species, including kiwifruit [4], the White Campion [5], and the Lesser Butterfly orchid [6]. Thus, fragrant lilac aldehydes represent an attractive olfactory target for insect pollinators. It is known that these flower-scenting molecules are much sought after by (nocturnal) moth species [7,8,9], butterflies [10,11], and even mosquitos [12,13,14]. In this context, lilac aldehydes are being developed as chemical markers of the botanical origin of honeys [15,16,17,18]. Interestingly, lilac aldehydes were recently found as odour-active compounds in oysters, and thus, could be potentially used as freshness indicators for this delicacy [19]. For perfumery purposes, synthetically prepared lilac aldehydes are used almost exclusively. Interestingly, the major naturally occurring (5′*S*)-stereoisomers **1a**–**d** have a lower odour threshold by 1–2 orders of magnitude in comparison to lilac aldehydes **1e**–**h** with (5′*R*)- absolute configuration [2] (Figure 1).

**Figure 2:**
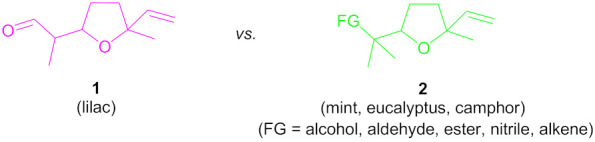
Comparison of olfactory profiles of lilac aldehydes **1** vs. methylated homologues **2**.

Up to date, numerous syntheses of racemic [4,8,20,21,22,23,24,25] and enantiomerically pure lilac aldehydes **1** are known [2,26,27,28]. Recently, we have published the first comprehensive study investigating the importance of respective substituents on the genuine flowery odour of lilac aldehydes. We have found [29] that the addition of methyl group to C-2 stereogenic centre of lilac aldehydes **1** significantly shifts the original flowery odour to a rather herbal scent of their unnatural homologues **2**. In addition, various functional groups have only minimal effect on the scent variations (Figure 2).

**Figure 3:**
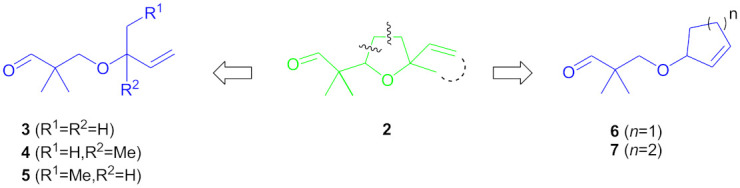
Structural design of racemic *seco*-analogues **3**–**7** of C-2 methylated lilac aldehydes **2**.

As a continuation of our research, we decided to investigate the influence of tetrahydrofuran ring and/or alkene moiety on the olfactory properties of C-2 methylated homologues of lilac aldehydes **2**. Thus, we have devised a new set of (racemic) *seco*-analogues **3**–**7** featuring a higher conformational freedom and/or variable substitution pattern of the C=C bond (Figure 3).

## 2. Results and Discussion

**Scheme 1:**
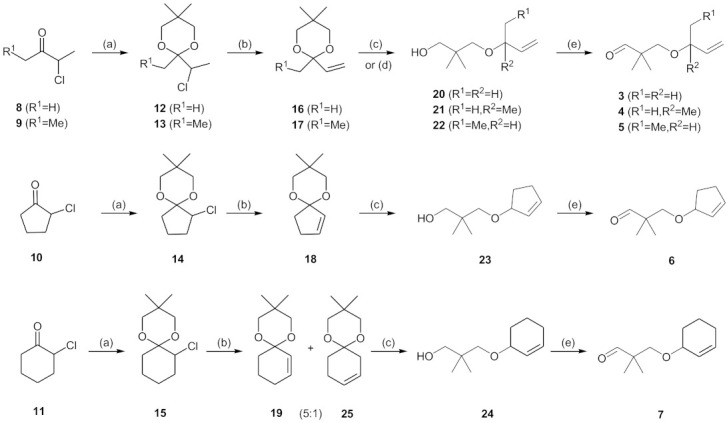
Preparation of *seco*-analogues **3**–**7**. Reagents and conditions: (a) cat. *p*-TsOH, 2,2-dimethyl-1,3-propanediol, cyclohexane, reflux (Dean–Stark), 5–27 h, crude **12** (90%), FLC or vacuum distillation, **13** (51%), **14** (82%), **15** (87%); (b) KOH (11 equiv), 1,2-ethanediol, 160 °C, 7–23 h, FLC or vacuum distillation, **16** (60%), **17** (71%), **18** (60%), **19** + **25** (5:1, 83%); (c) DIBAL (3–5 equiv), THF/toluene, 50 °C, 2–4 d, FLC or vacuum distillation, **20** (61%), **22** (15%), **23** (18%), **24** (48%); (d) MeMgBr (10 equiv), toluene/Et_2_O, 80 °C, 4 h, MPLC, **21** (23%); (e) Dess–Martin periodinane (1.10–1.25 equiv), NaHCO_3_ (0.27 equiv), DCM, RT, 2–6 h, FLC or PTLC, **3** (38%), **4** (39%), **5** (46%), **6** (22%), **7** (60%).

The preparation of novel *seco*-analogues **3**–**7** features a common synthetic strategy that starts with an acid-catalysed ketalisation of commercially available α-chloroketones **8**–**11** with neopentyl glycol followed by elimination of intermediary chloroketals **12**–**15** under basic conditions [30]. Unlike the chlorocyclopentane ketal **14**, the elimination of chlorocyclohexane ketal **15** provided an inseparable mixture of desired cyclohexene ketal **19** and its regioisomer **25** in a ratio of 5:1 (based on the integration of respective olefinic signals in ^1^H NMR spectrum). Thus, the obtained alkenyl ketals **16**–**19** were reductively opened either by diisobutyl aluminium hydride [31] or methyl magnesium bromide [32] to furnish the corresponding alcohols **20**–**24**. In case of DIBAL reduction of mixture of ketals **19** + **25**, only **19** was opened while **25** remained untouched under the reaction conditions. This enabled the efficient FLC separation of unreacted ketal **25** from desired alcohol **24**. Eventually, the final Dess–Martin oxidation [33] of pure alcohols **20**–**24** provided target aldehydes **3**–**7** in overall yields of 2–21% over four steps (Scheme 1).

With *seco*-analogues of lilac aldehydes **3**–**7** in our hands, we have undertaken their qualitative sensory evaluation. Since lilac alcohols also contribute to the complex scent of lilac flowers, we have also included their acyclic analogues **20**–**24** in the olfactory screening. The results are summarised in Table 1. Evidently, the opening of the tetrahydrofuran ring leads to a vanishing of flowery scent typical for lilac aldehydes **1**, suggesting the important olfactophoric role of THF moiety. Thus, ring-opened analogues **3**–**5** bearing an acyclic alkene exhibit a rather common spicy, sharp aroma with green/herbal notes. The scent of their congeners featuring a cyclic alkene is further shifted towards sweet, fruity aspects for **6** and herbal, green notes for **7**. Interestingly and for comparison, while the respective alcohols **20**, **22**, **23**, and **24** commonly exhibit sweet(ish) aroma with oily/fatty and turpentine-like facets, alcohol **21** features a pleasant balsamic aroma with minty-eucalypty notes. Finally, the odour intensity for target compounds was rather weak to moderate with only short aroma persistence in a range of tens of minutes.

## 3. Materials and Methods 

### 3.1. Materials and Methods

Chemicals and reagents were purchased from commercial sources (Alfa Aesar, Sigma–Aldrich) and were used without further purification. Anhydrous solvents were prepared by distillation and standing over activated 4Å molecular sieves under argon atmosphere. Hexanes refer to a mixture of C-6 alkanes (b.p. 60–80 °C). Yields refer to chromatographically and spectroscopically (^1^H NMR) homogeneous material, unless otherwise stated. Reactions were monitored by thin layer chromatography (TLC) carried out on aluminium sheets pre-coated with silica gel 60 F_254_ (Merck). Visualisation was performed using shortwave UV light followed by dipping TLC plates in either basic solution of KMnO_4_, acidic solution of vanillin or acidic solution of ceric ammonium nitrate followed by heating with a heat gun. Preparative thin layer chromatography (PTLC) was carried out on glass plates (20 × 20 cm) coated with PLC silica gel 60 (layer thickness 1 mm) with concentrating zone (20 × 4 cm). Flash column chromatography (FLC) was performed using Silica Gel 60 (particle size 40–63 μm). Medium-pressure liquid chromatography (MPLC) was performed on Büchi Sepacor System equipped with Pump Module C-615 and Fraction Collector C-660 using silica gel (particle size 15–40 μm). NMR spectra were recorded in CDCl_3_ on a Varian INOVA 300 (300 MHz for ^1^H, 75 MHz for ^13^C nuclei), Varian 400-MR (399.9 MHz for ^1^H, 100.6 MHz for ^13^C nuclei) or Varian VNMRS 600 (600 MHz for ^1^H, 151 MHz for ^13^C nuclei) NMR spectrometer and were correctly shifted using residual non-deuterated solvent as an internal reference (CHCl_3_: δ_H_ = 7.26 ppm, δ_C_ = 77.16 ppm (central peak of a 1:1:1 triplet). Chemical shifts (δ) are quoted in ppm. Liquid chromatography-mass spectrometry (LC-MS) analyses were performed on Agilent 1200 Series instrument equipped with a multimode MS detector using the MM ESI/APCI ionisation method (column Zorbax Eclipse XDB-18, 150 × 4.6 mm, particle size 5 μm, eluent water with 0.1% HCO_2_H/CH_3_CN, 70:30, flow 1.5 mL/min). High-resolution mass spectra (HR-MS) were recorded on a Thermo Scientific Orbitrap Velos mass spectrometer with a heated electrospray ionisation (HESI) source in positive and/or negative mode. FTIR spectra were obtained on a Nicolet 5700 spectrometer (Thermo Electron) equipped with a Smart Orbit (diamond crystal ATR) accessory using the reflectance technique (4000–400 cm^–1^). The sensory analysis was performed by authors in a clean and odourless environment at 22 °C by using testing strips of odourless absorbent paper. This was wetted with the tested compound and the paper strip was smelled at certain intervals, while the scent was recorded.

### 3.2. Synthetic Procedures and Analytical Data

#### 3.2.1. General Experiment for the Preparation of Chloroketals (**12**–**15**)

A stirred solution of chloroketone **8**–**11**, 2,2-dimethyl-1,3-propanediol (1.0 or 1.2 equiv) and *p*-TsOH·H_2_O (0.023 equiv) in cyclohexane (c = 0.7 M) was refluxed in a round bottom flask equipped with a Dean–Stark apparatus. After the indicated time, the mixture was cooled to RT and solvent was evaporated in vacuo (bath temperature 50–55 °C, pressure 30–50 mbar) and the resulting oil dissolved in Et_2_O. The ethereal solution was sequentially washed with sat. aq. NaHCO_3_ soln., water and brine, dried (MgSO_4_) and concentrated in vacuo (35 °C, 30 mbar). The obtained crude product was further used as such or purified either by bulb-to-bulb vacuum distillation (Kugelrohr) or FLC to furnish a corresponding chloroketal **12**–**15**. The structural characterization (^1^H-NMR, ^13^C-NMR spectra) of the new compound are presented in Supplementary Materials.

##### 2-(1-Chloroethyl)-2,5,5-trimethyl-[1,3]dioxane (**12**)

Prepared accordingly to Ref. [30]: commercial 3-chlorobutanone **8** (5.86 mL, 56 mmol), 2,2-dimethyl-1,3-propanediol (5.87 g, 56 mmol), *p*-TsOH·H_2_O (246 mg, 1.3 mmol), cyclohexane (81 mL), reflux (100 °C), 17 h, concentrated in vacuo (50 °C, 30 mbar), Et_2_O (61 mL), NaHCO_3_ (37 mL), water (37 mL), brine (37 mL), crude chloroketal **12** (9.79 g, 90%) as yellow oil; R_f_ (hexanes/AcOEt 5:1) 0.74; δ_H_ (300 MHz, CDCl_3_) 4.14 (q, *J* = 6.8 Hz, 1H, CHCl), 3.61 (dd, *J* = 11.5, 8.9 Hz, 2H, OCH_2_), 3.52–3.40 (m, 2H, OCH_2_), 1.53 (d, *J* = 6.8 Hz, 3H, Me), 1.46 (s, 3H, Me), 1.08 (s, 3H, Me_2_), 0.85 (s, 3H, Me_2_). NMR spectrum fully corresponds to the literature data [30]; *m/z* (ESI) 157.2 (100, M-Cl^+^), 158.0 (9%), 175.2 (76, M-Cl+H_2_O^+^), 176.0 (8%), t_R_ = 2.34 min.

##### 2-(1-Chloroethyl)-2-ethyl-5,5-dimethyl-[1,3]dioxane (**13**)

2-Chloropentan-3-one **9** (2.73 g, 23 mmol, prepared accordingly to Ref. [34]), 2,2-dimethyl-1,3-propanediol (2.36 g, 23 mmol), *p*-TsOH·H_2_O (99 mg, 0.52 mmol), cyclohexane (32.6 mL), reflux (130 °C), 27 h, concentrated in vacuo (50 °C, 45 mbar), Et_2_O (40 mL), NaHCO_3_ (30 mL), water (30 mL), brine (30 mL), FLC (176 g SiO_2_, Hex: Et_2_O 12:1, 650 mL), pure chloroketal **13** (2.41 g, 51%) as colourless oil; R_f_ (hexanes/AcOEt 6:1) 0.67; ν_max_ (ATR) 2956, 2869, 1469, 1396, 1186, 1142, 1126, 1093, 1081, 1038, 1016, 957, 916 cm^−1^; δ_H_ (600 MHz, CDCl_3_) 4.35 (q, *J* = 6.8 Hz, 1H, CHCl), 3.59 (d, *J* = 11.5 Hz, 1H, OCH_2_), 3.55 (d, *J* = 11.5 Hz, 1H, OCH_2_), 3.49 (d, *J* = 11.5 Hz, 1H, OCH_2_), 3.48 (d, *J* = 11.5 Hz, 1H, OCH_2_), 1.96 (m, 2H, CH_2_), 1.55 (d, *J =* 6.8 Hz, 3H, Me), 1.04 (s, 3H, Me_2_), 0.93 (t, *J* = 7.5 Hz, 3H, Me), 0.90 (s, 3H, Me_2_); δ_C_ (151 MHz, CDCl_3_) 100.1 (OC_q_), 70.2 (2 × OCH_2_), 56.5 (CHCl), 29.6 (C_q_), 22.9 (Me_2_), 22.5 (Me_2_), 21.2 (CH_2_), 18.7 (Me), 7.0 (Me); *m/z* (ESI) 189.2 (100, M-Cl+H_2_O^+^), 190.0 (10%), 171.2 (95, M-Cl^+^), 172.2 (8%), t_R_ = 2.96 min; HR-MS (HESI): M^+^, found 170.1305. C_10_H_18_O_2_ requires 170.1301.

##### 1-Chloro-8,8-dimethyl-6,10-dioxaspiro [4.5] decane (**14**)

Commercial 2-chlorocyclopentanone **10** (755 µL, 7.5 mmol), 2,2-dimethyl-1,3-propanediol (943 mg, 9.1 mmol), *p*-TsOH·H_2_O (33 mg, 0.17 mmol), cyclohexane (11 mL), reflux (128 °C), 4.5 h, concentrated in vacuo (50 °C, 50 mbar), Et_2_O (15 mL), NaHCO_3_ (6 mL), water (6 mL), brine (6 mL), FLC (41 g SiO_2_, Hex: AcOEt 6:1, 270 mL), pure chloroketal **14** (1.27 g, 82%) as colourless oil; R_f_ (hexanes/AcOEt 5:1) 0.67; ν_max_ (ATR) 2954, 2867, 1472, 1396, 1337, 1207, 1144, 1112, 1076, 1016, 837 cm^−1^; δ_H_ (300 MHz, CDCl_3_) 4.39–4.34 (m, 1H, CHCl), 3.55–3.45 (m, 4H, 2 × OCH_2_), 2.29–1.60 (m, 6H, 3xCH_2_), 1.00 (s, 3H, Me_2_), 0.98 (s, 3H, Me_2_); δ_C_ (151 MHz, CDCl_3_) 107.6 (OC_q_), 72.3 (OCH_2_), 72.1 (OCH_2_), 60.7 (CHCl), 32.9 (CH_2_), 31.1 (CH_2_), 30.0 (C_q_), 22.4 (Me_2_), 22.3 (Me_2_), 18.6 (CH_2_); HR-MS (HESI): M^+^, found 204.0913. C_10_H_17_ClO_2_ requires 204.0912.

##### 7-Chloro-3,3-dimethyl-1,5-dioxaspiro[5.5]undecane (**15**)

Commercial 2-chlorocyclohexanone **11** (860 µL, 7.5 mmol), 2,2-dimethyl-1,3-propanediol (942 mg, 9.0 mmol), *p*-TsOH·H_2_O (33 mg, 0.17 mmol), cyclohexane (10.9 mL), reflux (160 °C), 10 h, concentrated in vacuo (55 °C, 50 mbar), Et_2_O (20 mL), NaHCO_3_ (5 mL), water (5 mL), brine (5 mL), bulb-to-bulb distillation (175–180 °C, 27 mbar), pure chloroketal **15** (1.43 g, 87%) as yellow oil.; R_f_ (hexanes/AcOEt 5:1) 0.67; ν_max_ (ATR) 2942, 2864, 1472, 1446, 1395, 1364, 1215, 1167, 1112, 1111, 1096, 1016, 965, 804 cm^−1^; δ_H_ (600 MHz, CDCl_3_) 4.23–4.18 (m, 1H, CHCl), 3.62 (d, *J* = 11.4 Hz, 1H, OCH_2_), 3.58 (d, *J* = 11.5 Hz, 1H, OCH_2_), 3.52 (dd, *J* = 11.4, 1.8 Hz, 1H, OCH_2_), 3.45 (dd, *J* = 11.4, 1.8 Hz, 1H, OCH_2_), 2.33–2.23, 2.07–2.00, 1.96–1.88, 1.76–1.68 (4xm, 4 × 1H, 2 × CH_2_), 1.57–1.37 (m, 4H, 2 × CH_2_), 1.12 (s, 3H, Me_2_), 0.87 (s, 3H, Me_2_). ^1^H NMR spectrum fully corresponds the literature data [35]; δ_C_ (151 MHz, CDCl_3_) 97.2 (OC_q_), 70.3 (OCH_2_), 69.8 (OCH_2_), 61.6 (CHCl), 32.0 (C_q_, CH_2_), 30.0 (CH_2_), 27.4 (CH_2_), 22.8 (Me_2_), 22.4 (Me_2_), 21.6 (CH_2_); HR-MS (HESI): M^+^, found 218.1071. C_11_H_19_ClO_2_ requires 218.1068.

#### 3.2.2. General Experiment for the Preparation of Alkene Ketals (**16**–**19**, **25**)

To a stirred solution of potassium hydroxide (11 eq.) in ethylene glycol was added to chloroketal **12**–**15** at 120 °C and the reaction mixture was heated at 160 °C for 7–23 h. After cooling to RT, water was added and extracted with Et_2_O. The organic phase was washed with water and brine, dried (MgSO_4_) and concentrated in vacuo (35 °C, 30–200 mbar). The crude product was purified either by bulb-to-bulb vacuum distillation (Kugelrohr) or FLC to furnish a corresponding ketal **16**–**19**, **25**.

##### 2,5,5-Trimethyl-2-vinyl-[1,3]dioxane (**16**)

KOH (31.72 g, 0.57 mol), ethylene glycol (68 mL), chloroketal **12** (9.76 g, 51 mmol), 23 h, water (340 mL), Et_2_O (3 × 150 mL), water (110 mL), brine (110 mL), concentrated in vacuo (35 °C, 30 mbar), bulb-to-bulb vacuum distillation (80–95 °C, 27 mbar), ketal **16** (4.76 g, 60%) as colourless oil; R_f_ (hexanes/AcOEt 5:1) 0.72; δ_H_ (300 MHz, CDCl_3_) 5.76 (dd, *J* = 17.8, 10.8 Hz, 1H, C*H*=CH_2_), 5.38 (dd, *J* = 13.6, 1.5 Hz, 1H, CH=C*H*_2_), 5.33 (dd, *J* = 6.6, 1.5 Hz, 1H, CH=C*H*_2_), 3.58 (d, *J* = 10.6 Hz, 2H, OCH_2_), 3.32 (dt, *J* = 11.1, 1.2 Hz, 2H, OCH_2_), 1.40 (s, 3H, Me), 1.17 (s, 3H, Me_2_), 0.70 (s, 3H, Me_2_). NMR spectrum is in accordance with the literature data [30].

##### 2-Ethyl-5,5-dimethyl-2-vinyl-[1,3]dioxane (**17**)

KOH (7.46 g, 0.13 mol), ethylene glycol (16 mL), chloroketal **13** (2.46 g, 12 mmol), 21 h, water (200 mL), Et_2_O (3 × 80 mL), water (80 mL), brine (80 mL), concentrated in vacuo (35 °C, 200 mbar), FLC (112 g SiO_2_, DCM, 400 mL), ketal **17** (1.44 g, 71%) as colourless oil; R_f_ (DCM) 0.54; ν_max_ (ATR) 2952, 2870, 1471, 1403, 1395, 1363, 1179, 1154, 1085, 955, 914 cm^−1^; δ_H_ (600 MHz, CDCl_3_) 5.66 (dd, *J* = 17.7, 11.0 Hz, 1H, C*H*=CH_2_), 5.40 (dd, *J* = 11.0, 1.6 Hz, 1H, CH=C*H*_2_), 5.33 (dd, *J* = 17.8, 1.6 Hz, 1H, CH=C*H*_2_), 3.61 (d, *J* = 11.0 Hz, 2H, OCH_2_), 3.32 (d, *J* = 11.2 Hz, 2H, OCH_2_), 1.65 (q, *J* = 7.5 Hz, 2H, CH_2_), 1.17 (s, 3H, Me_2_), 0.92 (t, *J* = 7.5 Hz, 3H, Me), 0.69 (s, 3H, Me_2_); δ_C_ (151 MHz, CDCl_3_) 137.4 (*C*H=CH_2_), 119.0 (CH=*C*H_2_), 100.5 (OC_q_), 71.5 (2 × OCH_2_), 34.5 (CH_2_), 30.2 (C_q_), 22.8 (Me_2_), 22.0 (Me_2_), 7.1 (Me); *m/z* (ESI) 171.2 (82, M+H^+^), 172.2 (8%), t_R_ = 2.89 min; HR-MS (HESI): M^+^, found 170.1310. C_10_H_18_O_2_ requires 170.1301.

##### 8,8-Dimethyl-6,10-dioxaspiro[4.5]dec-1-ene (**18**)

KOH (3.88 g, 69 mmol), ethylene glycol (8.3 mL), chloroketal **14** (1.27 g, 6.2 mmol), 7 h, water (90 mL), Et_2_O (3 × 50 mL), water (50 mL), brine (50 mL), concentrated in vacuo (35 °C, 30 mbar), bulb-to-bulb vacuum distillation (125–130 °C, 28 mbar), ketal **18** (0.62 g, 60%) as yellow oil; R_f_ (hexanes/AcOEt 6:1) 0.51; ν_max_ (ATR) 2952, 2855, 1473, 1395, 1363, 1203, 1141, 1129, 1088, 1048, 1036, 955, 908, 894 cm^−1^; δ_H_ (600 MHz, CDCl_3_) 6.16 (dt, *J* = 5.8, 2.2 Hz, 1H, C*H*=CH_2_), 6.10 (dt, *J* = 5.9, 2.4 Hz, 1H, CH=C*H*_2_), 3.59 (d, *J* = 11.3 Hz, 2H, OCH_2_), 3.53 (d, *J* = 11.3 Hz, 2H, OCH_2_), 2.42 (dt, *J* = 7.5, 6.7, 2.3 Hz, 2H, CH_2_), 2.14–2.11 (m, 2H, CH_2_), 1.01 (s, 3H, Me_2_), 0.98 (s, 3H, Me_2_); δ_C_ (151 MHz, CDCl_3_) 136.9 (*C*H=CH_2_), 128.9 (CH=*C*H_2_), 111.9 (OC_q_), 72.5 (2 × OCH_2_), 33.4 (CH_2_), 30.0 (C_q_), 29.6 (CH_2_), 22.6 (Me_2_), 22.4 (Me_2_); HR-MS (HESI): M^+^, found 168.1144. C_10_H_16_O_2_ requires 168.1145.

##### 3,3-Dimethyl-1,5-dioxaspiro[5.5]undec-7-ene (**19**) and 3,3-Dimethyl-1,5-dioxaspiro[5.5]undec-8-ene (**25**)

KOH (2.86 g, 51 mmol), ethylene glycol (6.1 mL), chloroketal **15** (1.0 g, 4.6 mmol), 18.5 h, water (60 mL), Et_2_O (3 × 20 mL), water (20 mL), brine (20 mL), concentrated in vacuo (35 °C, 30 mbar), bulb-to-bulb vacuum distillation (145–155 °C, 27 mbar) to give a mixture of ketals **19**:**25** in ratio 5:1 determined by ^1^H NMR spectroscopy (0.69 g, 83%) as colourless oil; R_f_ (hexanes/AcOEt 5:1) 0.59.

Ketal (**19**): δ_H_ (600 MHz, CDCl_3_) 6.10 (dt, *J* = 10.4, 2.1 Hz, 1H, C*H*=CH_2_), 5.92 (dt, *J* = 10.3, 3.6 Hz, 1H, C*H*=CH_2_), 3.62 (d, *J* = 11.4 Hz, 2H, OCH_2_), 3.51 (d, *J* = 11.4 Hz, 2H, OCH_2_), 2.07–2.03 (m, 2H, CH_2_), 1.93–1.90 (m, 2H, CH_2_), 1.75–1.70 (m, 2H, CH_2_), 1.02 (s, 3H, Me_2_), 0.95 (s, 3H, Me_2_); δ_C_ (151 MHz, CDCl_3_) 132.1 (*C*H=CH_2_), 125.2 (CH=*C*H_2_), 95.1 (OC_q_), 70.4 (2 × OCH_2_), 32.0 (CH_2_), 30.1 (C_q_), 25.6 (CH_2_), 22.8 (Me_2_), 22.6 (Me_2_), 19.3 (CH_2_); *m/z* (ESI) 183.0 (53, M+H^+^), 184.0 (7%), t_R_ = 2.44 min.

Ketal (**25**): ν_max_ (ATR) 3027, 2952, 2861, 1470, 1361, 1269, 1251, 1105, 1036, 1015, 845 cm^−1^; δ_H_ (600 MHz, CDCl_3_) 5.70–5.66 (m, 1H, C*H*=CH_2_), 5.58–5.54 (m, 1H, C*H*=CH_2_), 3.59 (d, *J* = 11.5 Hz, 2H, OCH_2_), 3.49 (d, *J* = 11.5 Hz, 2H, OCH_2_), 2.39–2.35 (m, 2H, CH_2_), 2.16–2.11 (m, 2H, CH_2_), 1.96 (t, *J* = 6.5 Hz, 2H, CH_2_), 1.03 (s, 3H, Me_2_), 0.92 (s, 3H, Me_2_); δ_C_ (151 MHz, CDCl_3_) 126.4 (*C*H=CH_2_), 123.4 (CH=*C*H_2_), 97.0 (OC_q_), 70.2 (2 × OCH_2_), 34.8 (CH_2_), 30.2 (C_q_), 27.3 (CH_2_), 23.5 (CH_2_), 22.8 (Me_2_), 22.5 (Me_2_); HR-MS (HESI): M^+^, found 183.1316. C_11_H_18_O_2_ requires 182.1301.

#### 3.2.3. Preparation of Alcohol (**21**)

##### 2,2-Dimethyl-3-(2-methyl-but-3-en-2-yloxy)propan-1-ol (**21**)

To a stirred solution of ketal **16** (0.3 g, 1.9 mmol) in anhydrous toluene (32 mL) was added 3M solution of MeMgBr in diethyl ether (6.4 mL, 19.2 mmol, 10 equiv) drop-wise at 0 °C and the mixture was heated at 80 °C for 4 h under Ar. After cooling the mixture to 0 °C, the reaction was carefully quenched with sat. aq. NH_4_Cl soln. (25 mL) and water (10 mL). The layers were separated and the aqueous phase was extracted with Et_2_O (3 × 15 mL). The combined organic layers were washed with brine (30 mL) and dried over Na_2_SO_4_. The solvents were evaporated in vacuo (75 °C, 29 mbar) and crude material was purified by MPLC(16 g SiO_2_, flow 50 mL/min, fraction 5 mL, gradient elution hexanes/AcOEt 90:10 → 50:50) to furnish alcohol **21** (75 mg, 23%) as colourless oil; R_f_ (hexanes/AcOEt 5:1) 0.37; ν_max_ (ATR) 3433 (OH), 2976, 2870, 1475, 1415, 1360, 1150, 1076, 1044, 999, 920, 875 cm^−1^; δ_H_ (400 MHz, CDCl_3_) 5.68 (dd, *J* = 17.8, 10.6 Hz, 1H, C*H*=CH_2_), 5.13 (dd, *J* = 4.4, 1.1 Hz, 1H, CH=C*H*_2_), 5.10 (dd, *J* = 2.6, 1.1 Hz, 1H, CH=C*H*_2_), 3.42 (d, *J* = 5.0 Hz, 2H, CH_2_), 3.17 (brs, 3H, CH_2_, OH), 1.25 (s, 6H, Me_2_), 0.89 (s, 6H, Me_2_); δ_C_ (101 MHz, CDCl_3_) 143.5 (*C*H=CH_2_), 114.1 (CH=*C*H_2_), 75.2 (OC_q_), 72.7 (CH_2_), 72.6 (CH_2_), 35.5 (C_q_), 25.6 (Me_2_), 22.0 (Me_2_); HR-MS (HESI): M^+^, found 172.1466. C_10_H_20_O_2_ requires 172.1463.

#### 3.2.4. General Experiment for the Preparation of Alcohols (**20**, **22**, **23**)

To a stirred solution of ketal **16**–**18,** anhydrous THF was added dropwise to a solution of DIBAL in toluene (1.0 M, 3–5 equiv) at 0 °C over 5–10 min under Ar. The mixture was warmed to 50 °C and stirred for 2–4.5 d. The cooled mixture (ice) was quenched with sat. aq. soln. of Rochelle salt and water, Et_2_O was added and stirred for 2–5 h at RT. The layers were separated and the aqueous phase was extracted with Et_2_O. The combined organic layers were washed with brine and dried over MgSO_4_. The solvents were evaporated in vacuo (73 °C, 30 mbar). The crude product was purified either by bulb-to-bulb vacuum distillation (Kugelrohr) or FLC to furnish a corresponding alcohol **20**, **22**, **23**.

##### 3-(But-3-en-2-yloxy)-2,2-dimethylpropan-1-ol (**20**)

Ketal **16** (0.5 g, 3.2 mmol), THF (16 mL), 1M DIBAL in toluene (17.4 mL, 17.4 mmol, 5.5 equiv, 7 min), 50 °C, 44 h, sat. aq. Rochelle salt soln. (25 mL), water (5 mL), Et_2_O (150 mL), 5 h, Et_2_O (2 × 25 mL), brine (50 mL), bulb-to-bulb vacuum distillation (135–145 °C, 31 mbar), alcohol **20** (307 mg, 61%) as colourless oil; R_f_ (hexanes/AcOEt 5:1) 0.3; ν_max_ (ATR) 3399 (OH), 2972, 2956, 2870, 1475, 1421, 1368, 1319, 1094, 1044, 991, 921 cm^−1^; δ_H_ (300 MHz, CDCl_3_) 5.70 (ddd, *J* = 17.3, 10.3, 7.1 Hz, 1H, C*H*=CH_2_), 5.17 (ddd, *J* = 14.6, 1.6, 1.0 Hz, 1H, CH=C*H*_2_), 5.12 (ddd, *J* = 7.6, 1.6, 1.0 Hz, 1H, CH=C*H*_2_), 3.82–3.71 (m, 1H, CH), 3.44 (s, 2H, CH_2_), 3.34 (d, *J* = 8.9 Hz, 1H, CH_2_), 3.16 (d, *J* = 8.8 Hz, 1H, CH_2_), 2.91 (brs, 1H, OH), 1.23 (d, *J* = 6.4 Hz, 3H, Me), 0.91 (s, 6H, Me_2_); δ_C_ (151 MHz, CDCl_3_) 140.0 (*C*H=CH_2_), 115.9 (CH=*C*H_2_), 78.2 (CH_2_), 77.8 (CH), 72.4 (CH_2_), 35.9 (C_q_), 21.9 (Me_2_), 21.2 (Me); HR-MS (HESI): M^+^, found 158.1303. C_9_H_18_O_2_ requires 158.1301.

##### 2,2-Dimethyl-3-(pent-1-en-3-yloxy)propan-1-ol (**22**)

Ketal **17** (0.8 g, 4.7 mmol), THF (23.5 mL), 1M DIBAL in toluene (25.9 mL, 25.9 mmol, 5.5 equiv, 5 min), 50 °C, 4.5 d, sat. aq. Rochelle salt soln. (42 mL), water (30 mL), Et_2_O (80 mL), 2 h, Et_2_O (2 × 40 mL), brine (80 mL), FLC (14.6 g SiO_2_, Hex/AcOEt 7:1, 180 mL), alcohol **22** (122 mg, 15%) as colourless oil; R_f_ (hexanes/AcOEt 5:1) 0.43; ν_max_ (ATR) 3411 (OH), 2961, 2873, 1475, 1422, 1322, 1091, 1045, 993, 921 cm^−1^; δ_H_ (600 MHz, CDCl_3_) 5.64 (ddd, *J* = 17.2, 10.5, 7.5 Hz, 1H, C*H*=CH_2_), 5.20–5.14 (m, 2H, CH=C*H*_2_), 3.50 (dd, *J* = 13.6, 6.7 Hz, 1H, CH), 3.44 (d, *J* = 5.2 Hz, 2H, OCH_2_), 3.38 (d, *J* = 8.8 Hz, 1H, OCH_2_), 3.11 (d, *J* = 8.8 Hz, 1H, OCH_2_), 2.91 (t, *J* = 5.6 Hz, 1H, OH), 1.63–1.47 (m, 2H, CH_2_), 0.93 (s, 3H, Me), 0.90 (s, 3H, Me), 0.90 (t, *J* = 7.5 Hz, 3H, Me); δ_C_ (151 MHz, CDCl_3_) 138.6 (*C*H=CH_2_), 116.9 (CH=*C*H_2_), 83.5 (OCH), 78.4 (OCH_2_), 72.4 (OCH_2_), 36.0 (C_q_), 28.3 (CH_2_), 21.9 (Me_2_), 9.6 (Me); HR-MS (HESI): M^+^, found 172.1463. C_10_H_20_O_2_ requires 172.1458.

##### 3-(Cyclopent-2-enyloxy)-2,2-dimethylpropanol (**23**)

Ketal **18** (596 mg, 3.5 mmol), THF (17.7 mL), 1M DIBAL in toluene (10.6 mL, 10.6 mmol, 3 equiv, 10 min), 50 °C, 50 h, sat. aq. Rochelle salt soln. (13 mL), water (20 mL), Et_2_O (60 mL), 2 h, Et_2_O (2 × 30 mL), brine (60 mL), FLC (27.9 g SiO_2_, gradient elution Hex/AcOEt 8:1 (360 mL) → 1:1 (100 mL)), alcohol **23** (107 mg, 18%) as colourless oil; R_f_ (hexanes/AcOEt 5:1) 0.29; ν_max_ (ATR) 3419 (OH), 2953, 2854, 1474, 1359, 1116, 1082, 1041 cm^−1^; δ_H_ (600 MHz, CDCl_3_) 6.02–5.99 (m, 1H, C*H*=CH), 5.82–5.79 (m, 1H, CH=C*H*), 4.53–4.50 (m, 1H, CH), 3.43 (s, 2H, OCH_2_), 3.32 (d, *J* = 8.7 Hz, 1H, OCH_2_), 3.29 (d, *J* = 8.7 Hz, 1H, OCH_2_), 2.91 (brs, 1H, OH), 2.50–2.43 (m, 1H, CH_2_), 2.28–2.22 (m, 1H, CH_2_), 2.16–2.09 (m, 1H, CH_2_), 1.77–1.70 (m, 1H, CH_2_), 0.91 (s, 6H, Me_2_); δ_C_ (151 MHz, CDCl_3_) 135.9 (*C*H=CH), 130.4 (CH=*C*H), 85.4 (CH), 78.1 (OCH_2_), 72.4 (OCH_2_), 35.9 (C_q_), 31.0 (CH_2_), 29.6 (CH_2_), 22.0 (Me_2_); *m/z* (ESI) 169.2 (100, M-H^+^), 170.2 (10, M^+^), t_R_ = 1.96 min; HR-MS (HESI): M^+^, found 170.1312. C_10_H_18_O_2_ requires 170.1301.

#### 3.2.5. Preparation of Alcohol (**24**)

To a cooled mixture of ketals **19** + **25** (5:1, 754 mg, 4.1 mmol) in anhydrous THF (20.7 mL) 1M solution of DIBAL in toluene (18.6 mL, 18.6 mmol, 4.5 equiv) was added dropwise at 0 °C over 5 min under Ar. The mixture was warmed to 50 °C and stirred for 88 h. The cooled mixture (ice) was quenched with sat. aq. soln. of Rochelle salt (30 mL) and water (15 mL), diluted with Et_2_O (70 mL) and stirred for 2.5 h at RT. The layers were separated and the aqueous phase was extracted with Et_2_O (2 × 35 mL). The combined organic layers were washed with brine (70 mL) and dried over MgSO_4_. The solvents were evaporated in vacuo (73 °C, 30 mbar). The crude material was purified by FLC (31 g SiO_2_, gradient elution DCM/^i^PrOH 70:1 (213 mL) → 35:1 (36 mL)) to furnish desired alcohol **24** (303 mg, 48%) and unreacted ketal **25** (75 mg) as colourless oils.

3-(Cyclohex-2-enyloxy)-2,2-dimethylpropanol (**24**): R_f_ (DCM/^i^PrOH 70:1) 0.19; ν_max_ (ATR) 3426 (OH), 3026, 2934, 2865, 1474, 1393, 1080, 1045, 947, 898, 725 cm^−1^; δ_H_ (300 MHz, CDCl_3_) 5.84–5.81 (dtd, *J* = 10.1, 3.5, 1.2 Hz, 1H, C*H*=CH), 5.74 (ddt, *J* = 10.1, 3.2, 2.0 Hz, 1H, CH=C*H*), 3.83–3.75 (m, 1H, CH), 3.44 (d, *J* = 3.8 Hz, 2H, OCH_2_), 3.38 (d, *J* = 8.7 Hz, 1H, OCH_2_), 3.33 (d, *J* = 8.6 Hz, 1H, OCH_2_), 2.99 (brs, 1H, OH), 2.10–1.45 (m, 6H, 3 × CH_2_), 0.92 (s, 6H, Me_2_); δ_C_ (151 MHz, CDCl_3_) 131.0 (*C*H=CH), 127.3 (CH=*C*H), 78.1 (OCH_2_), 73.5 (CH), 72.4 (OCH_2_), 36.0 (C_q_), 28.2 (CH_2_), 25.1 (CH_2_), 21.9 (Me_2_), 19.1 (CH_2_); *m/z* (ESI) 105.2 (100%), 185.2 (58, M+H^+^), 186.2 (10%), t_R_ = 3.13 min; HR-MS (HESI): M^+^, found 184.1445. C_11_H_20_O_2_ requires 184.1458.

#### 3.2.6. General Experiment for the Preparation of Aldehydes (**3**–**7**)

To a solution of alcohol **20**–**24** in anhydrous DCM Dess–Martin periodinane (DMP, 1.10–1.25 equiv) and NaHCO_3_ (0.27 equiv) was added under Ar. The mixture was stirred for 2–6 h at RT and quenched with sat. aq. NaHCO_3_ soln. followed by addition of sat. aq. Na_2_S_2_O_3_ soln. and the stirring continued until all solids dissolved (1–4 h). The layers were separated, the aqueous layer was extracted with DCM and the combined organic layers were washed with brine and dried over MgSO_4_. The solvent was removed in vacuo (40 °C, 100–200 mbar) and residue was purified by either FLC or PTLC to furnish corresponding aldehyde **3**–**7**.

##### 3-(But-3-en-2-yloxy)-2,2-dimethylpropan-1-al (**3**)

Alcohol **20** (163 mg, 1.03 mmol), DCM (10.3 mL), DMP (546 mg, 1.29 mmol, 1.25 equiv), NaHCO_3_ (24 mg, 0.28 mmol), 3 h, sat. aq. NaHCO_3_ soln. (8.4 mL), sat. aq. Na_2_S_2_O_3_ soln. (8.4 mL), 1 h, DCM (3 × 25 mL), brine (40 mL), evaporation in vacuo (40 °C, 200 mbar), FLC (8.5 g SiO_2_, DCM, 150 mL), aldehyde **3** (62 mg, 38%) as colourless oil; R_f_ (DCM) 0.5; ν_max_ (ATR) 2974, 2932, 2871, 1729 (C=O), 1474, 1367, 1092, 992, 925, 910 cm^−1^; δ_H_ (600 MHz, CDCl_3_) 9.55 (s, 1H, CHO), 5.68 (ddd, *J* = 17.3, 10.3, 7.1 Hz, 1H, C*H*=CH_2_), 5.15 (dt, *J* = 17.2, 1.3 Hz, 1H, CH=C*H*_2_), 5.11 (dt, *J* = 10.3, 1.4 Hz, 1H, CH=C*H*_2_), 3.78–3.72 (m, 1H, CH), 3.46 (d, *J* = 9.3 Hz, 1H, CH_2_), 3.29 (d, *J* = 9.3 Hz, 1H, CH_2_), 1.19 (d, *J* = 6.4 Hz, 3H, Me), 1.06 (s, 6H, 2 × Me); δ_C_ (151 MHz, CDCl_3_) 205.7 (CHO), 140.1 (*C*H=CH_2_), 115.8 (CH=*C*H_2_), 77.6 (CH), 73.4 (CH_2_), 47.0 (C_q_), 21.0 (Me), 19.0 (Me_2_); *m/z* (ESI) 157.2 (26, M+H^+^), 158.2 (3%), t_R_ = 2.48 min; HR-MS (HESI): M^+^, found 156.1139. C_9_H_16_O_2_ requires 156.1145.

##### 2,2-Dimethyl-3-(2-methyl-but-3-en-2-yloxy)propan-1-al (**4**)

Alcohol **21** (81 mg, 0.47 mmol), DCM (4.7 mL), DMP (219 mg, 0.52 mmol), NaHCO_3_ (11 mg, 0.13 mmol), 2.5 h, sat. aq. NaHCO_3_ soln. (3.8 mL, sat. aq. Na_2_S_2_O_3_ soln. (3.8 mL), 1 h, DCM (3 × 12 mL), brine (25 mL), evaporation in vacuo (40 °C, 200 mbar), FLC (6.3 g SiO_2_, Hexanes/Et_2_O 8:1, 180 mL), aldehyde **4** (31 mg, 39%) as colourless oil; R_f_ (hexanes/Et_2_O 8:1) 0.55; ν_max_ (ATR) 2976, 1731 (C=O), 1361, 1150, 1080, 1002, 924 cm^−1^; δ_H_ (400 MHz, CDCl_3_) 9.52 (s, 1H, CHO), 5.75 (dd, *J* = 17.8, 10.6 Hz, 1H, C*H*=CH_2_), 5.12 (dd, *J* = 4.9, 1.2 Hz, 1H, CH=C*H*_2_), 5.08 (dd, *J* = 2.2, 1.2 Hz, 1H, CH=C*H*_2_), 3.28 (s, 2H, CH_2_), 1.22 (s, 6H, 2 × Me), 1.04 (s, 6H, 2 × Me); δ_C_ (100 MHz, CDCl_3_) 206.1 (CHO), 143.7 (*C*H=CH_2_), 113.9 (CH=*C*H_2_), 74.9 (OC_q_), 67.8 (CH_2_), 46.7 (C_q_), 25.5 (Me_2_), 19.0 (Me_2_); HR-MS (HESI): M^+^, found 170.1309. C_10_H_18_O_2_ requires 170.1301.

##### 2,2-Dimethyl-3-(pent-1-en-3-yloxy)propan-1-al (**5**)

Alcohol **22** (107 mg, 0.62 mmol), DCM (6.2 mL), DMP (328 mg, 0.77 mmol), NaHCO_3_ (14 mg, 0.17 mmol), 3 h, sat. aq. NaHCO_3_ soln. (5 mL), sat. aq. Na_2_S_2_O_3_ soln. (5 mL), 1.5 h., DCM (3 × 15 mL), brine (30 mL), evaporation in vacuo (40 °C, 100 mbar), FLC (8.8 g SiO_2_, Hexanes/Et_2_O 7:1, 80 mL), aldehyde **5** (48 mg, 46%) as colourless oil; R_f_ (hexanes/Et_2_O 5:1) 0.61; ν_max_ (ATR) 2996, 1731, 1454, 1322, 1092, 924 cm^−1^; δ_H_ (600 MHz, CDCl_3_) 9.56 (s, 1H, CHO), 5.62 (ddd, *J* = 17.0, 10.6, 7.5 Hz, 1H, C*H*=CH_2_), 5.17–5.13 (m, 2H, CH=C*H*_2_), 3.50 (d, *J* = 9.3 Hz, 1H, OCH_2_), 3.51–3.46 (m, 1H, CH), 3.24 (d, *J* = 9.3 Hz, 1H, OCH_2_), 1.59–1.51 (m, 1H, CH_2_), 1.50–1.42 (m, 1H, CH_2_), 1.07 (s, 6H, 2 x Me), 0.86 (t, *J* = 7.4 Hz, 3H, Me); δ_C_ (151 MHz, CDCl_3_) 205.7 (CHO), 138.8 (*C*H=CH_2_), 116.7 (CH=*C*H_2_), 83.4 (CH), 73.7 (OCH_2_), 47.2 (C_q_), 28.2 (CH_2_), 19.0 (Me_2_), 9.6 (Me); HR-MS (HESI): M^+^, found 170.1305. C_10_H_18_O_2_ requires 170.1301.

##### 3-(Cyclopent-2-enyloxy)-2,2-dimethylpropan-1-al (**6**)

Alcohol **23** (87 mg, 0.51 mmol), DCM (5.1 mL), DMP (270 mg, 0.64 mmol), NaHCO_3_ (12 mg, 0.14 mmol), 2 h, sat. aq. NaHCO_3_ soln. (4.2 mL), sat. aq. Na_2_S_2_O_3_ soln. (4.2 mL), 2 h, DCM (3 × 13 mL), brine (30 mL), evaporation in vacuo (40 °C, 200 mbar), PTLC (Hexanes/Et_2_O 3:1), aldehyde **6** (19 mg, 22%) as colourless oil; R_f_ (hexanes/Et_2_O 3:1) 0.61; ν_max_ (ATR) 2931, 2854, 1729 (C=O), 1359, 1116, 1087, 1043, 897, 731 cm^−1^; δ_H_ (600 MHz, CDCl_3_) 9.55 (s, 1H, CHO), 6.01–5.98 (m, 1H, C*H*=CH), 5.82–5.79 (m, 1H, CH=C*H*), 4.55–4.51 (m, 1H, CH), 3.44 (d, *J* = 9.2 Hz, 1H, OCH_2_), 3.42 (d, *J* = 9.2 Hz, 1H, OCH_2_), 2.48–2.41 (m, 1H, CH_2_), 2.28–2.21 (m, 1H, CH_2_), 2.14–2.07 (m, 1H, CH_2_), 1.75–1.68 (m, 1H, CH_2_), 1.07 (s, 6H, 2 × Me); δ_C_ (151 MHz, CDCl_3_) 205.8 (CHO), 135.7 (*C*H=CH), 130.5 (CH=*C*H), 85.4 (CH), 73.3 (OCH_2_), 47.0 (C_q_), 31.1 (CH_2_), 29.4 (CH_2_), 19.1 (Me_2_); HR-MS (HESI): M^+^, found 168.1141. C_10_H_16_O_2_ requires 168.1145.

##### 3-(Cyclohex-2-enyloxy)-2,2-dimethylpropan-1-al (**7**)

Alcohol **24** (150 mg, 0.81 mmol), DCM (8.1 mL), DMP (432 mg, 1.02 mmol), NaHCO_3_ (19 mg, 0.23 mmol), 5.5 h, sat. aq. NaHCO_3_ soln. (6.6 mL), sat. aq. Na_2_S_2_O_3_ soln. (6.6 mL), 3.5 h, DCM (3 × 20 mL), brine (30 mL), evaporation in vacuo (40 °C, 100 mbar), FLC (7.9 g SiO_2_, DCM, 180 mL), aldehyde **7** (89 mg, 60%) as colourless oil; R_f_ (DCM) 0.56; ν_max_ (ATR) 2933, 2865, 1729 (C=O), 1474, 1395, 1084, 948, 895, 726 cm^−1^; δ_H_ (600 MHz, CDCl_3_) 9.57 (s, 1H, CHO), 5.85–5.81 (m, 1H, C*H*=CH), 5.73–5.69 (m, 1H, CH=C*H*), 3.82–3.77 (m, 1H, CH), 3.50 (d, *J* = 9.1 Hz, 1H, OCH_2_), 3.46 (d, *J* = 9.1 Hz, 1H, OCH_2_), 2.06–1.99 (m, 1H, CH_2_), 1.97–1.90 (m, 1H, CH_2_), 1.82–1.76 (m, 1H, CH_2_), 1.75–1.68 (m, 1H, CH_2_), 1.65–1.58 (m, 1H, CH_2_), 1.55–1.48 (m, 1H, CH_2_), 1.08 (s, 3H, Me), 1.07 (s, 3H, Me); δ_C_ (151 MHz, CDCl_3_) 205.8 (CHO), 130.8 (*C*H=CH), 127.5 (CH=*C*H), 73.6 (CH), 73.3 (OCH_2_), 47.1 (C_q_), 28.1 (CH_2_), 25.2 (CH_2_), 19.2 (CH_2_), 19.1 (Me_2_); HR-MS (HESI): M^+^, found 182.1302. C_11_H_18_O_2_ requires 182.1301.

## 4. Conclusions

We have designed, synthesised, and evaluated a set of new (racemic) *seco*-analogues **3**–**7** of lilac aldehydes featuring a higher conformational freedom and/or variable substitution pattern of the alkene moiety. Thus, starting from commercially available α-chloroketones **8**–**11**, we have prepared target aldehydes from intermediary alcohols **20**–**24** via the reductive ring-opening of respective ketals **16**–**19**. The qualitative olfactory analysis of novel *seco*-analogues **3**–**7** revealed that the opening of tetrahydrofuran ring leads to a vanishing of flowery scent typical for lilac aldehydes **1**, suggesting the important osmophoric role of THF moiety in these natural products. Moreover, while the ring-opened analogues **3**–**5** bearing an acyclic alkene exhibit a rather common spicy aroma with green notes, the scent of their congeners featuring a cyclic alkene is further shifted towards fruity aspects for **6** and herbal notes for **7**. For comparison purposes, we have also included alcohols **20**–**24** in the olfactory screening. Interestingly, while the respective alcohols **20**, **22**, **23**, and **24** commonly exhibit sweet aroma with fatty and turpentine-like facets, alcohol **21** features a pleasant balsamic aroma with minty-eucalypty notes. However, the aroma intensity for all compounds was weak to moderate with only short persistence over time.

## Figures and Tables

**Table 1:** Olfactory properties of compounds **3**–**7**, **20**–**24** prepared via Scheme 1.

Alcohol	Aroma	Aldehyde	Aroma
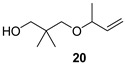	Sweet, herbal, minty, eucalypty	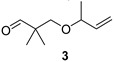	Spicy, terpenic, pungent
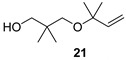	Balsamic, pleasant, minty, eucalypty	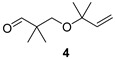	Spicy, camphor, sharp, with green notes
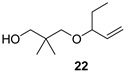	Oily, sweetish, turpentine-like	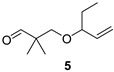	Herbal, green, sweetish, ethereal, sharp
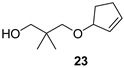	Fruity-sweetish, fatty, turpentine	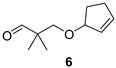	Sweet, fruity, with eucalyptus notes
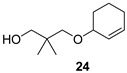	Bitter-sweet, turpentine-like	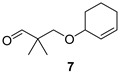	Herbal, slightly green, with earthy notes

## Data Availability

Not applicable.

## Supplementary Materials

The copies of NMR spectra of new compounds are available online.

## Sample Availability

Samples of the compounds are not available from the authors.
