# Supplementary material for: Synthesis and Olfactory Properties of Seco-Analogues of Lilac Aldehydes
Source: Molecules. 2021 Nov 23;26(23):7086. doi: 10.3390/molecules26237086 (PMC8658798; doi:10.3390/molecules26237086)
Supplement: Supplementary file 1 [file molecules-26-07086-s001.zip › molecules-1429725-supplementary.pdf]

SUPPORTING INFORMATION

Synthesis and Olfactory Properties of *seco*-Analogues of Lilac Aldehydes

Vladimír Dacho and Peter Szolcsányi*

Department of Organic Chemistry, Slovak University of Technology, Radlinského 9, SK-812 37 Bratislava, Slovakia

CONTENTS:

Copies of NMR spectra of new compounds Pages 2-16

* Corresponding author. Tel.: +421-2-593-25745; e-mail: peter.szolcsanyi@stuba.sk

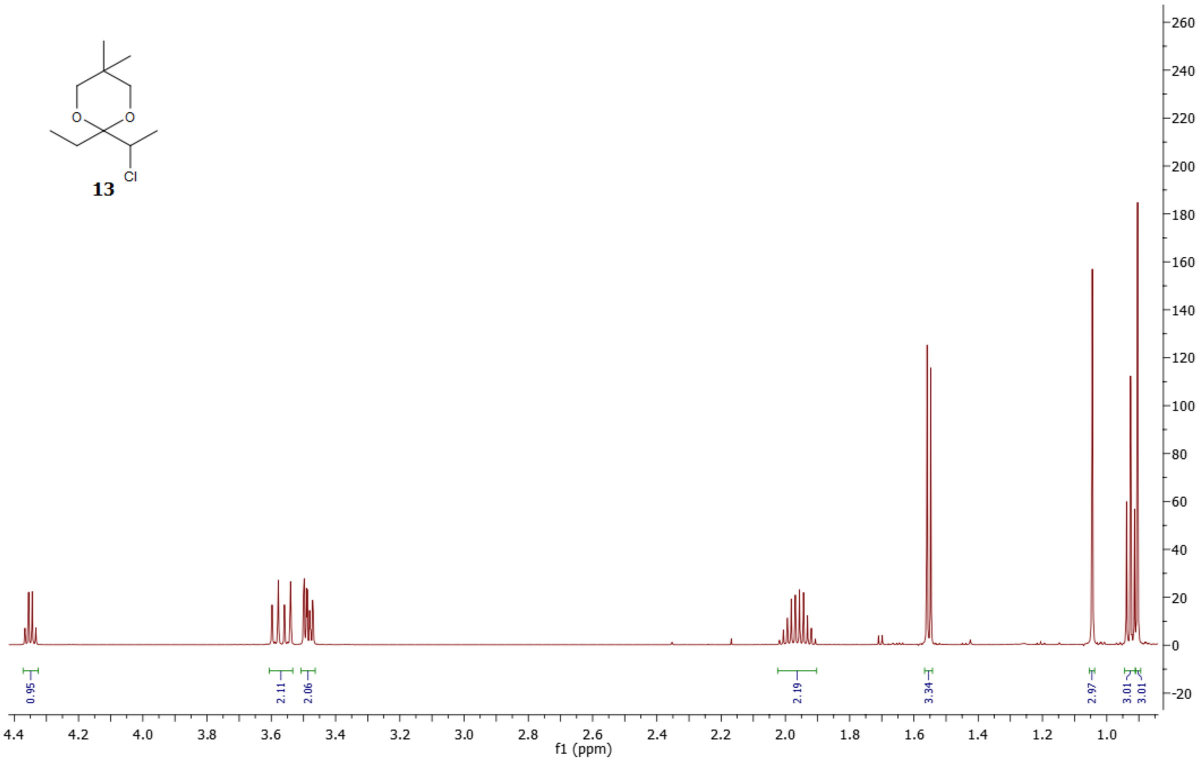
13
O
O
Cl
0.95
2.11
2.06
2.19
3.34
2.97
3.01
3.01
f1 (ppm)

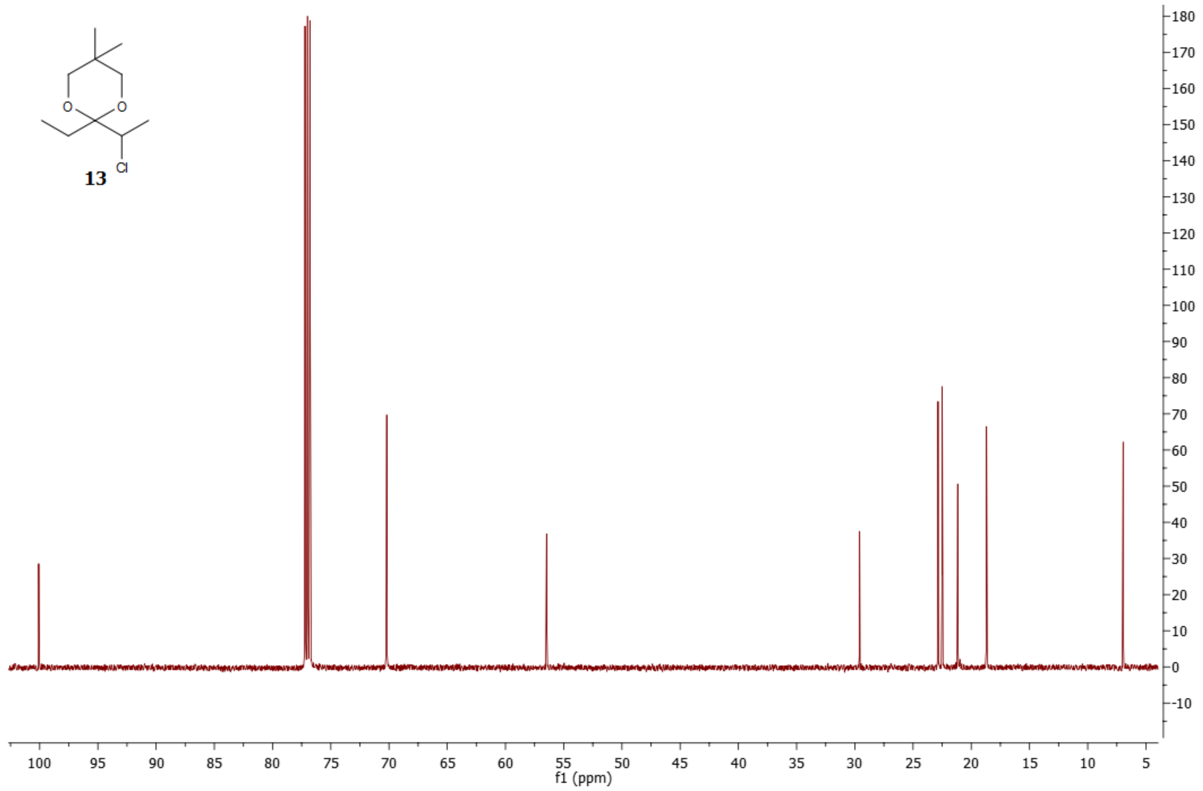
13
O
O
Cl
f1 (ppm)

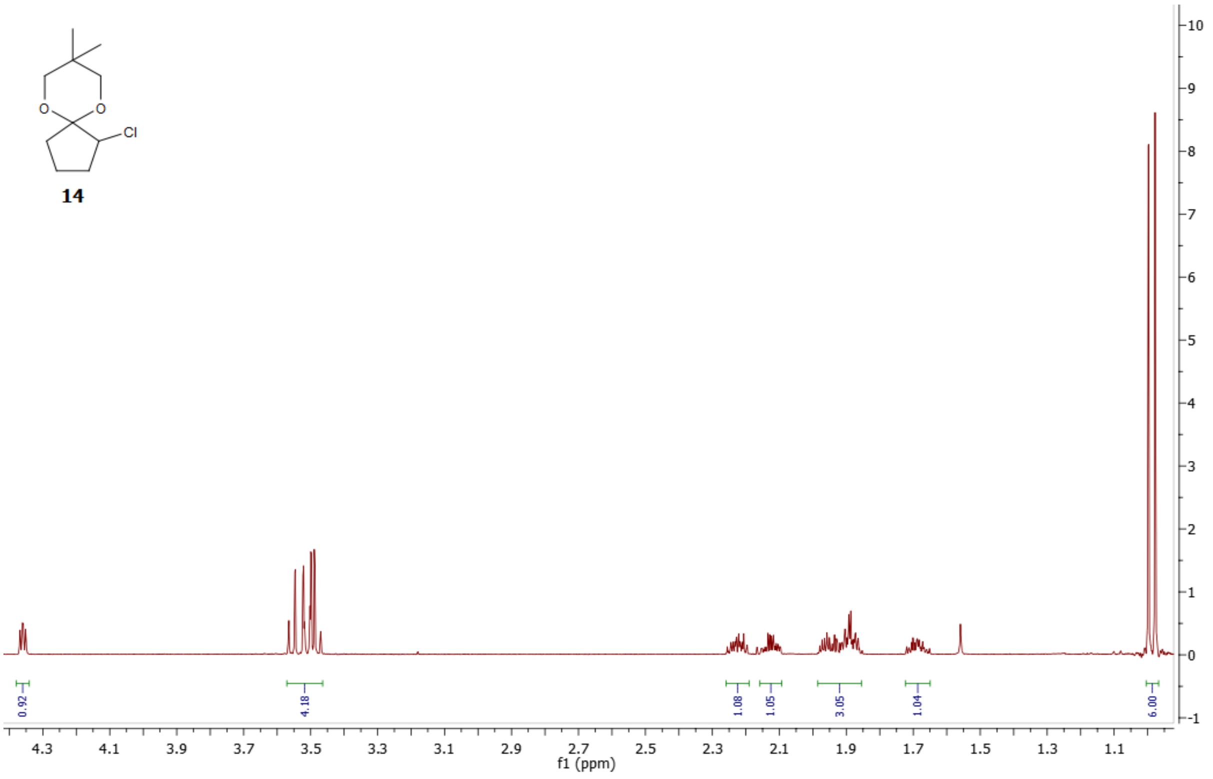
14
f1 (ppm)

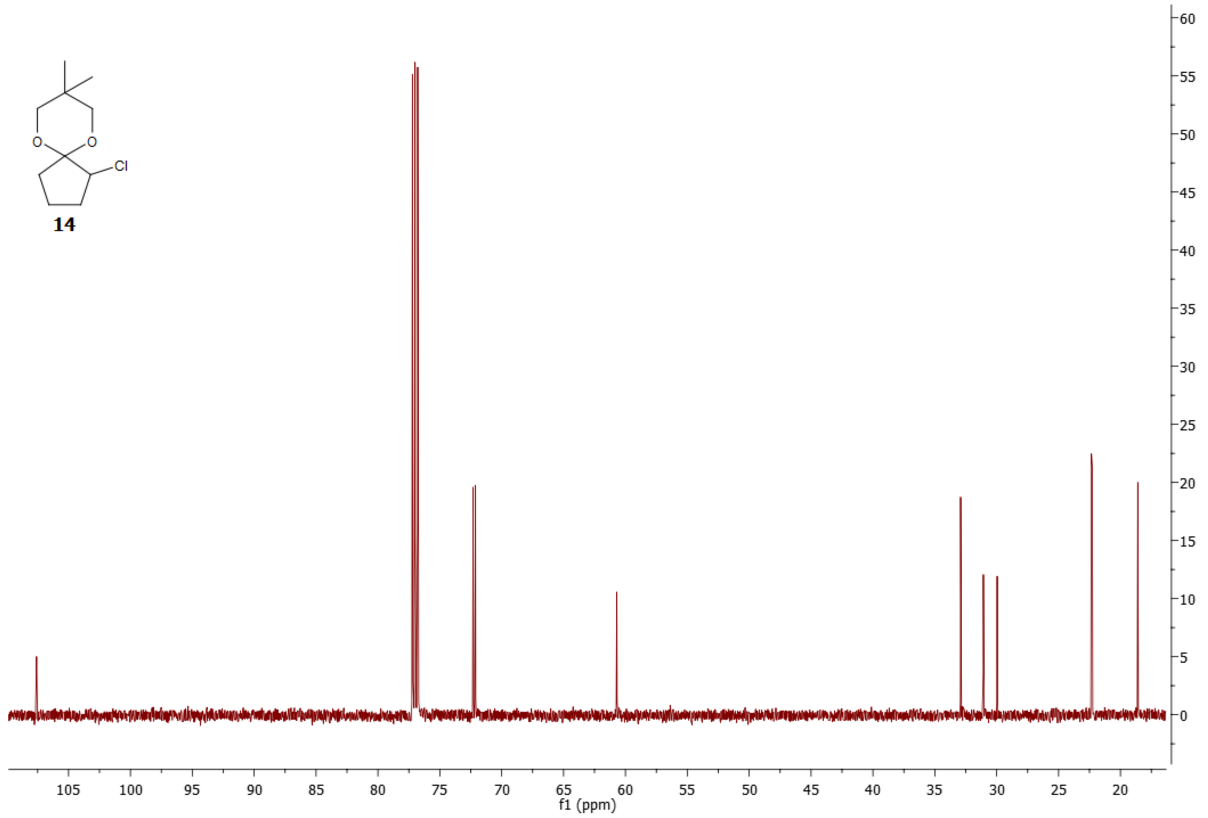
14
f1 (ppm)

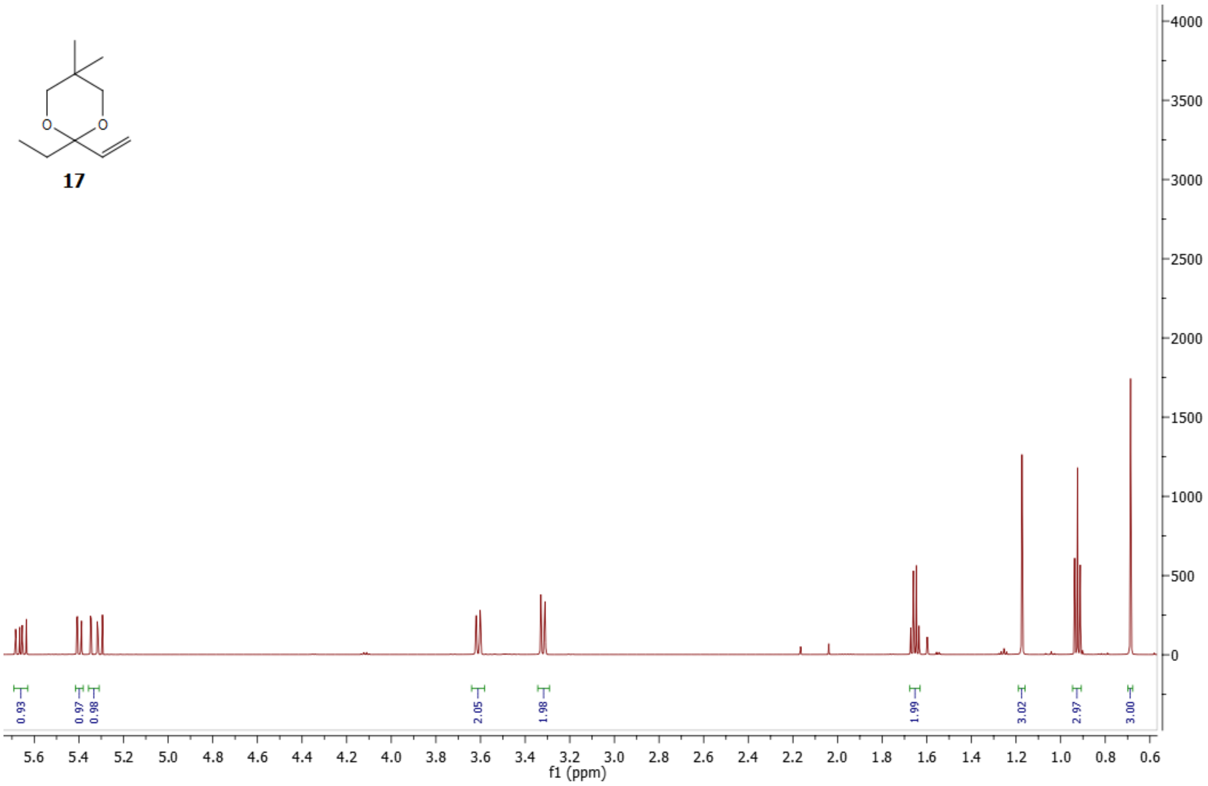
17
0.93
0.97
0.98
2.05
1.98
1.99
3.02
2.97
3.00
f1 (ppm)

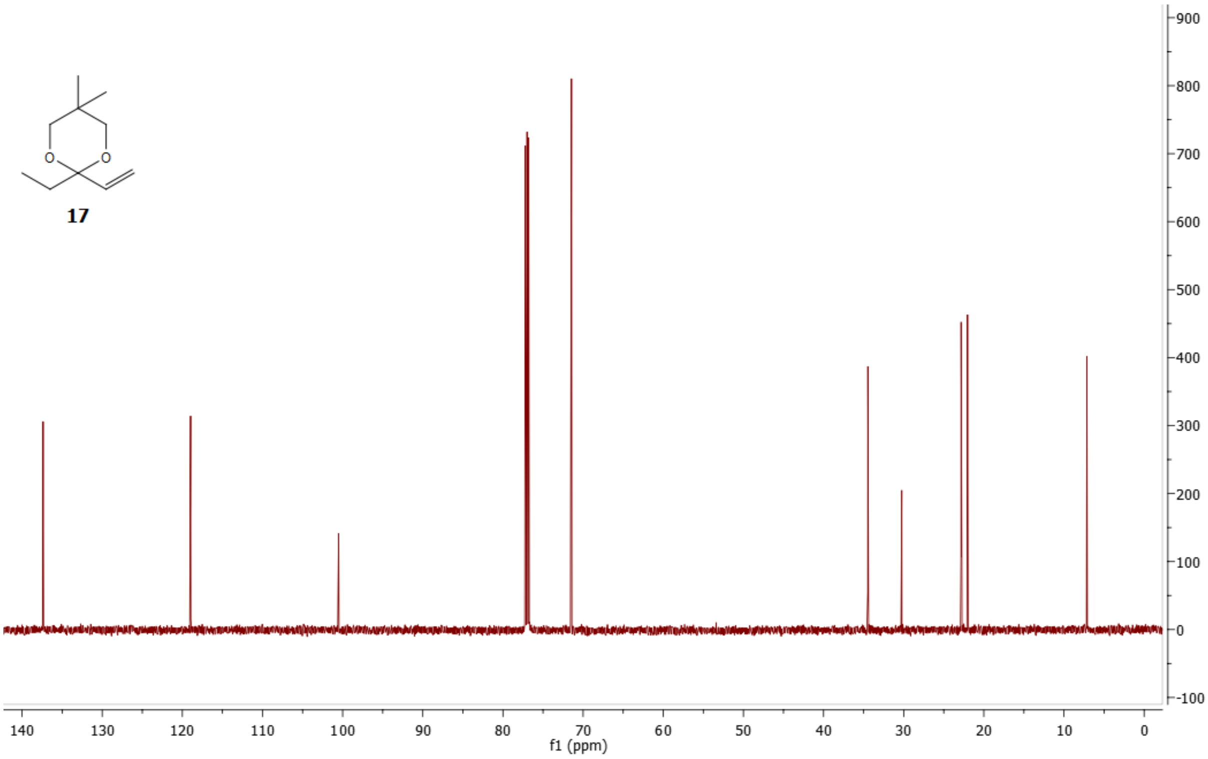
17
f1 (ppm)

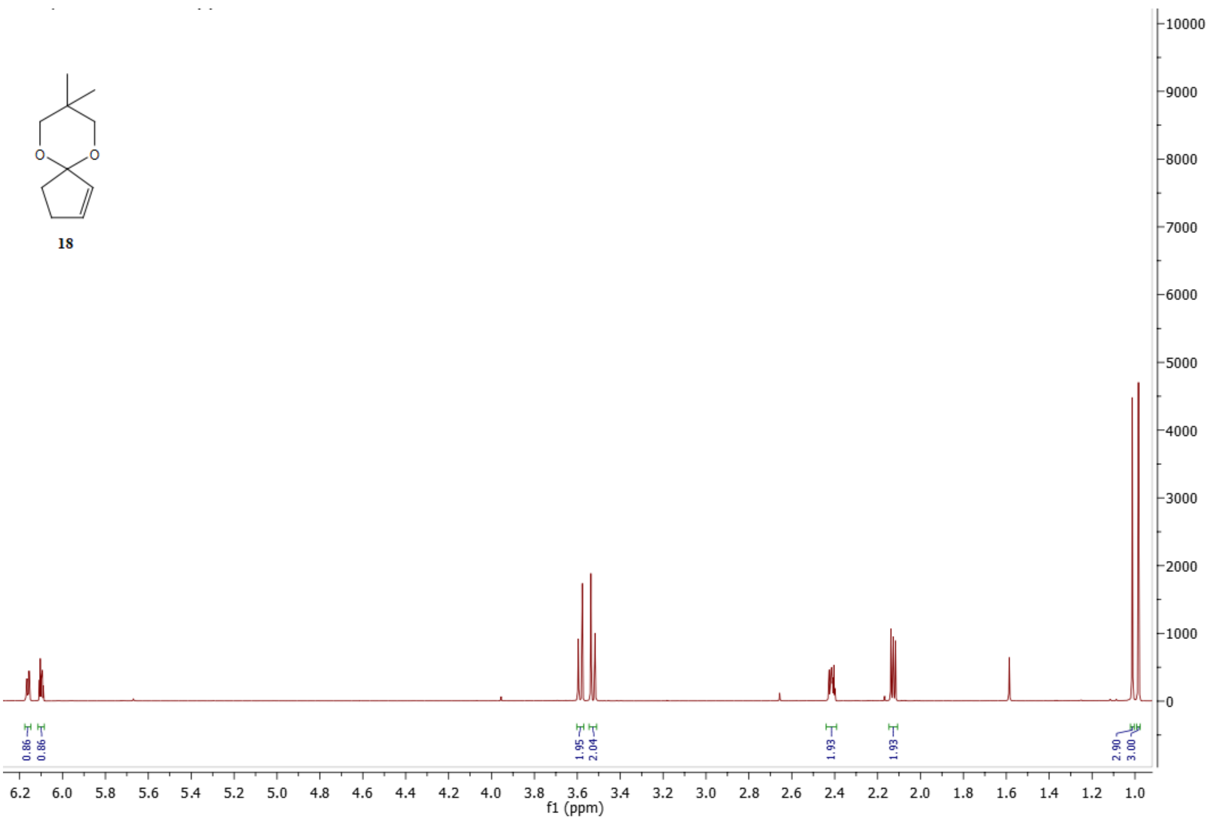
18
0.86
0.86
1.95
2.04
1.93
1.93
2.90
3.00
f1 (ppm)

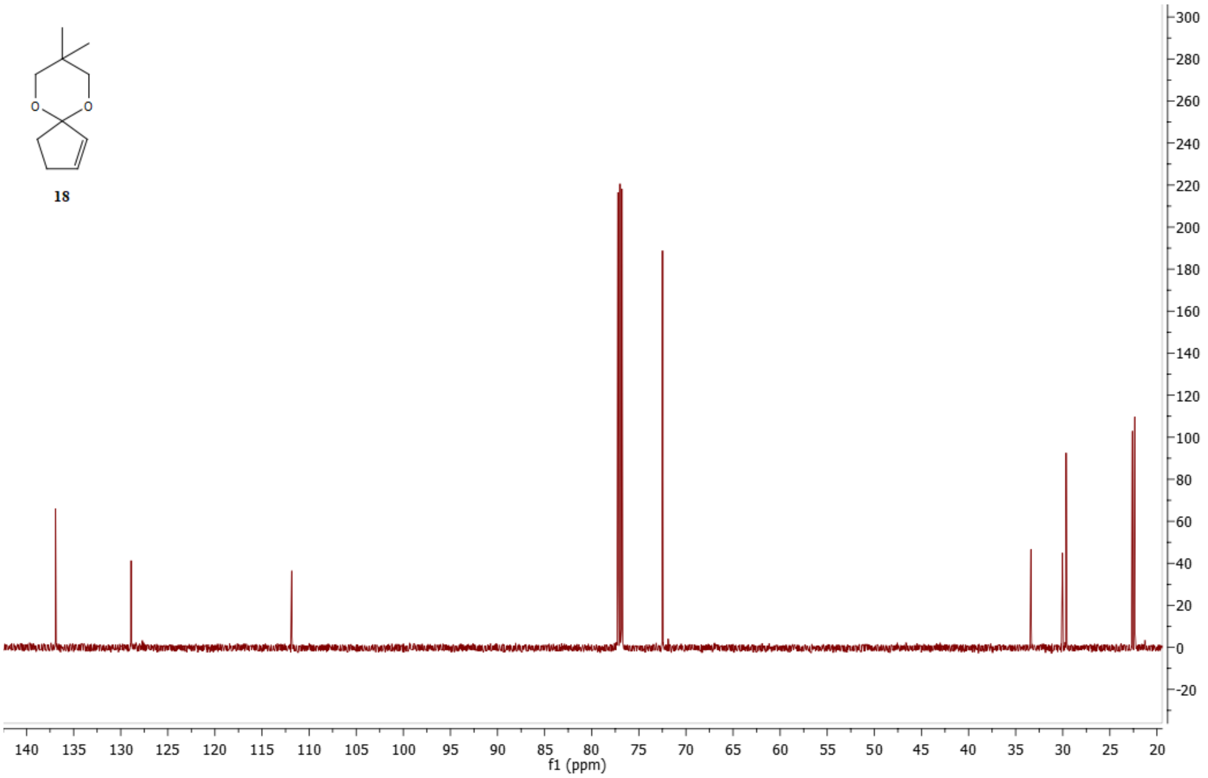
18
f1 (ppm)

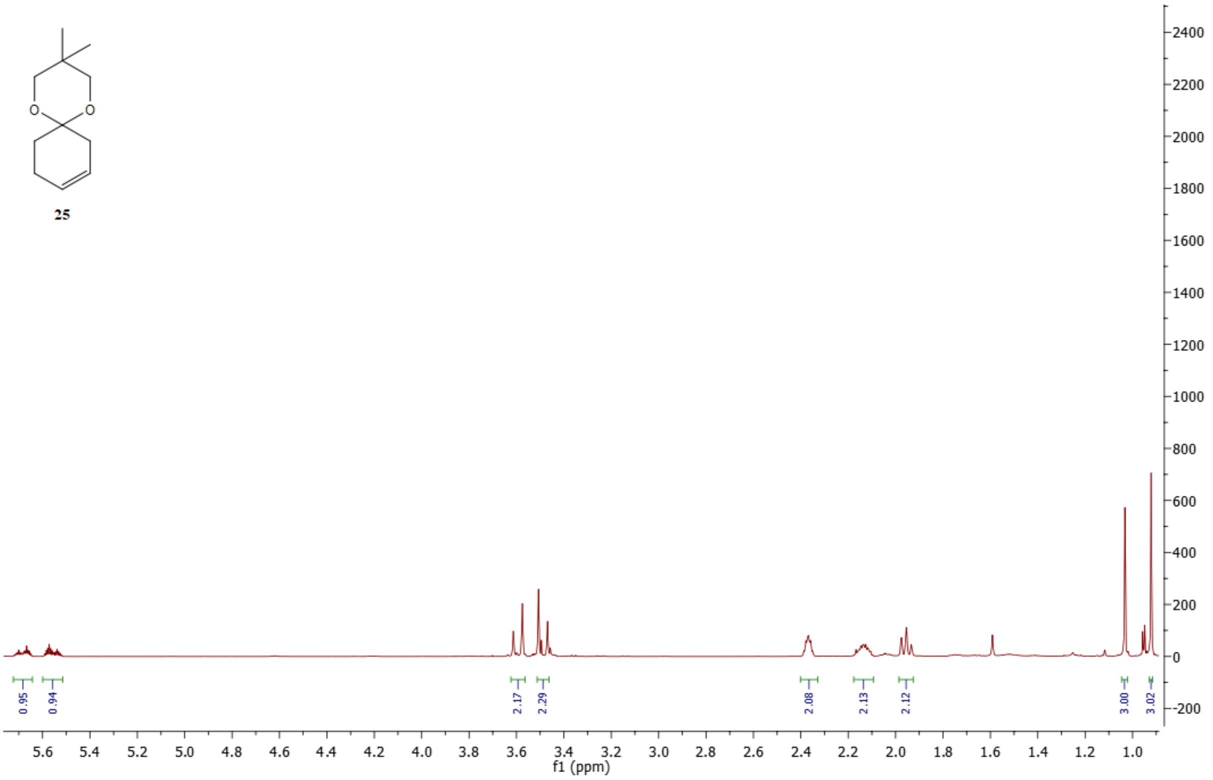
25
f1 (ppm)

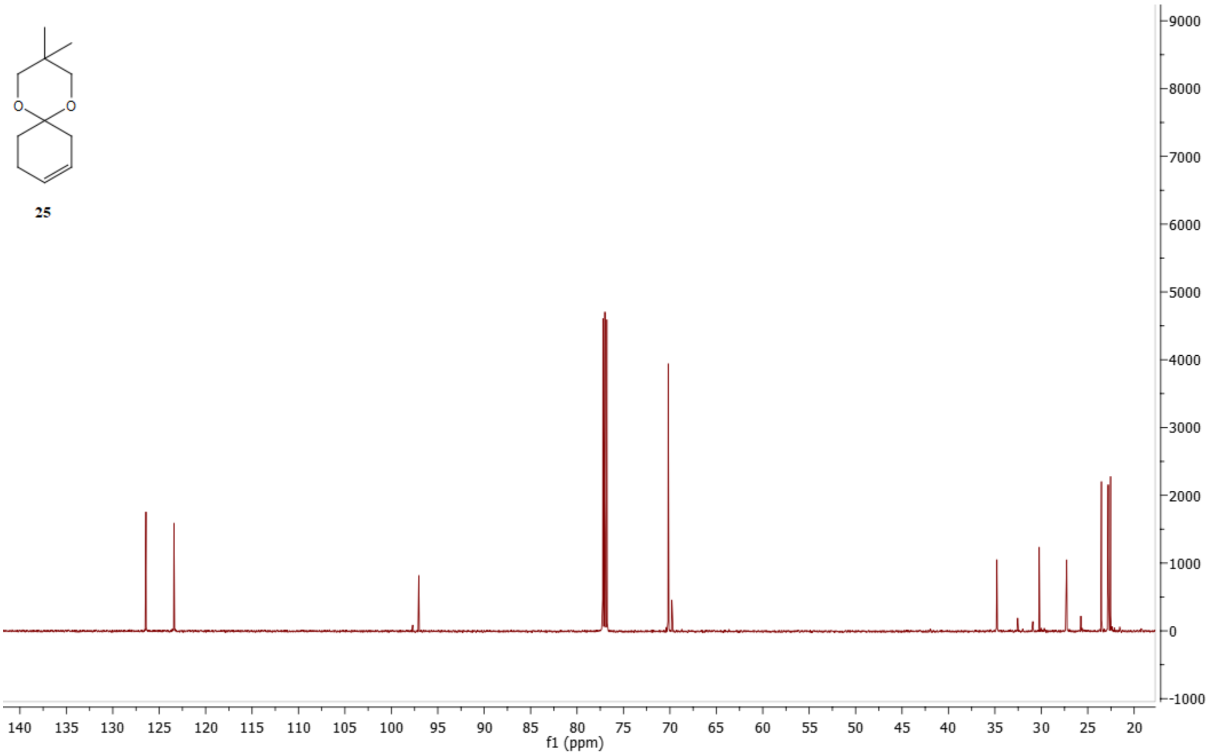
25
f1 (ppm)

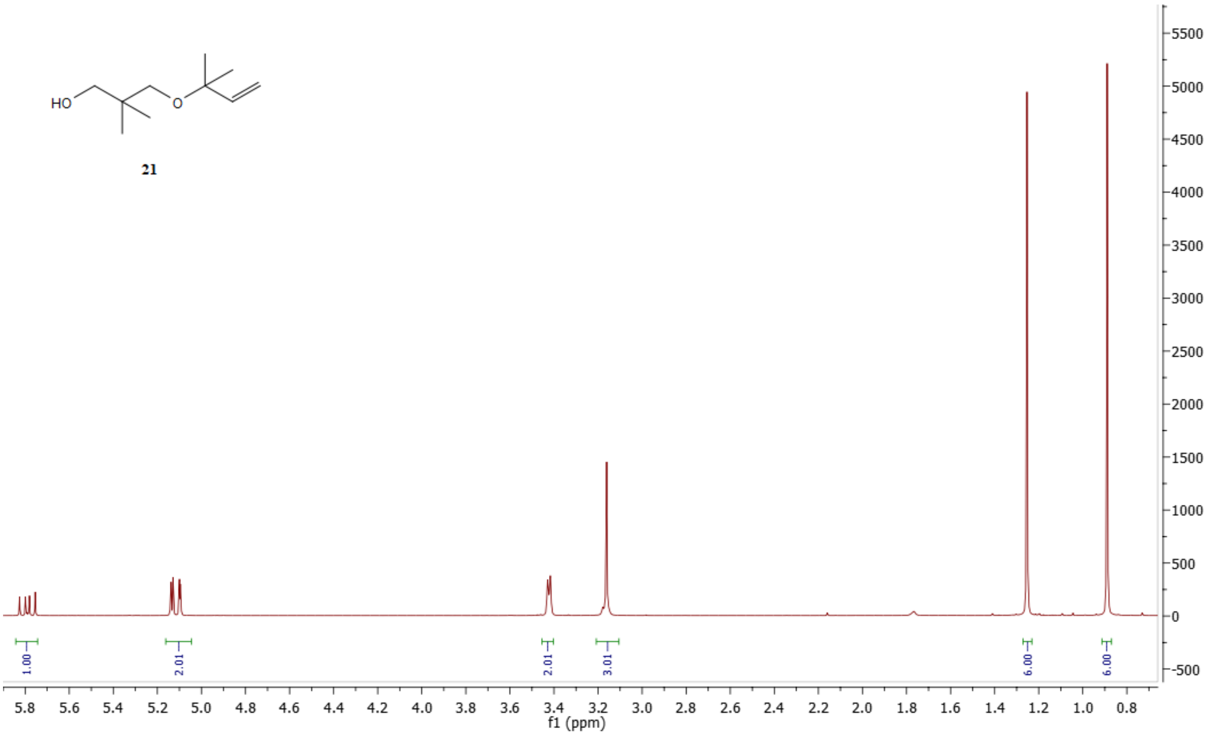
HO
O
21
1.00
2.01
2.01
3.01
6.00
6.00
f1 (ppm)

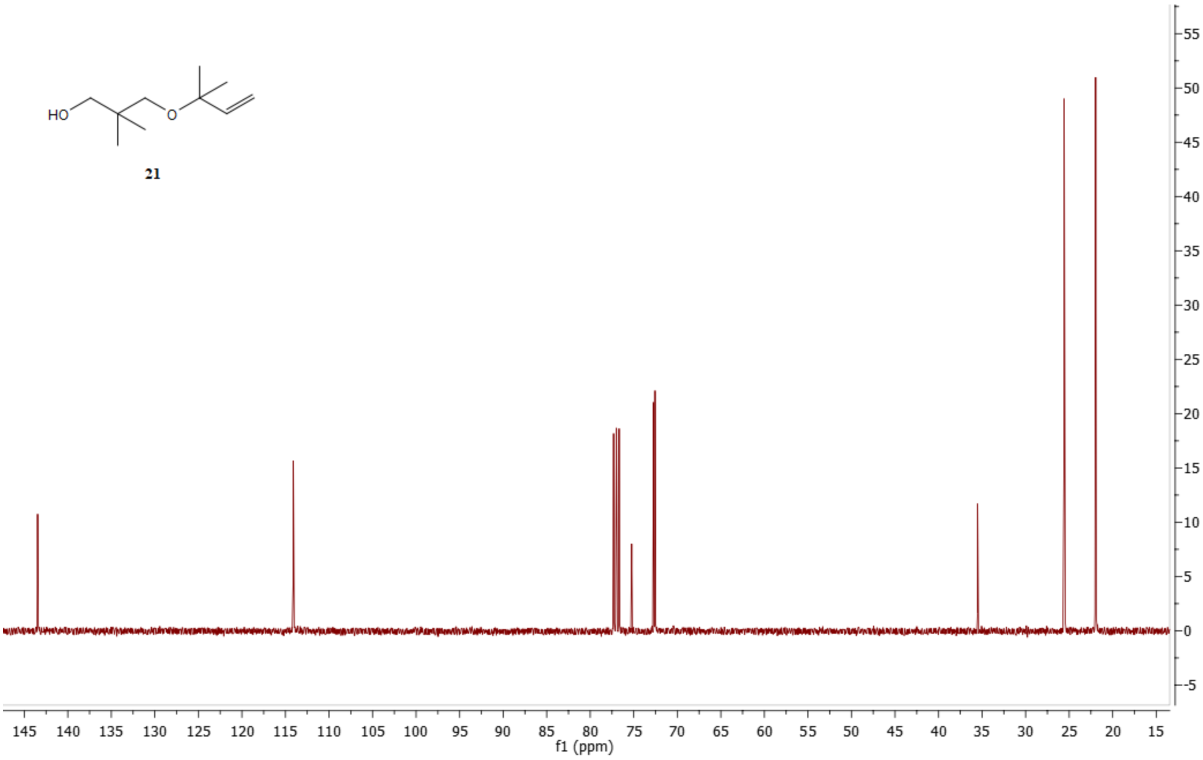
HO
O
21
f1 (ppm)

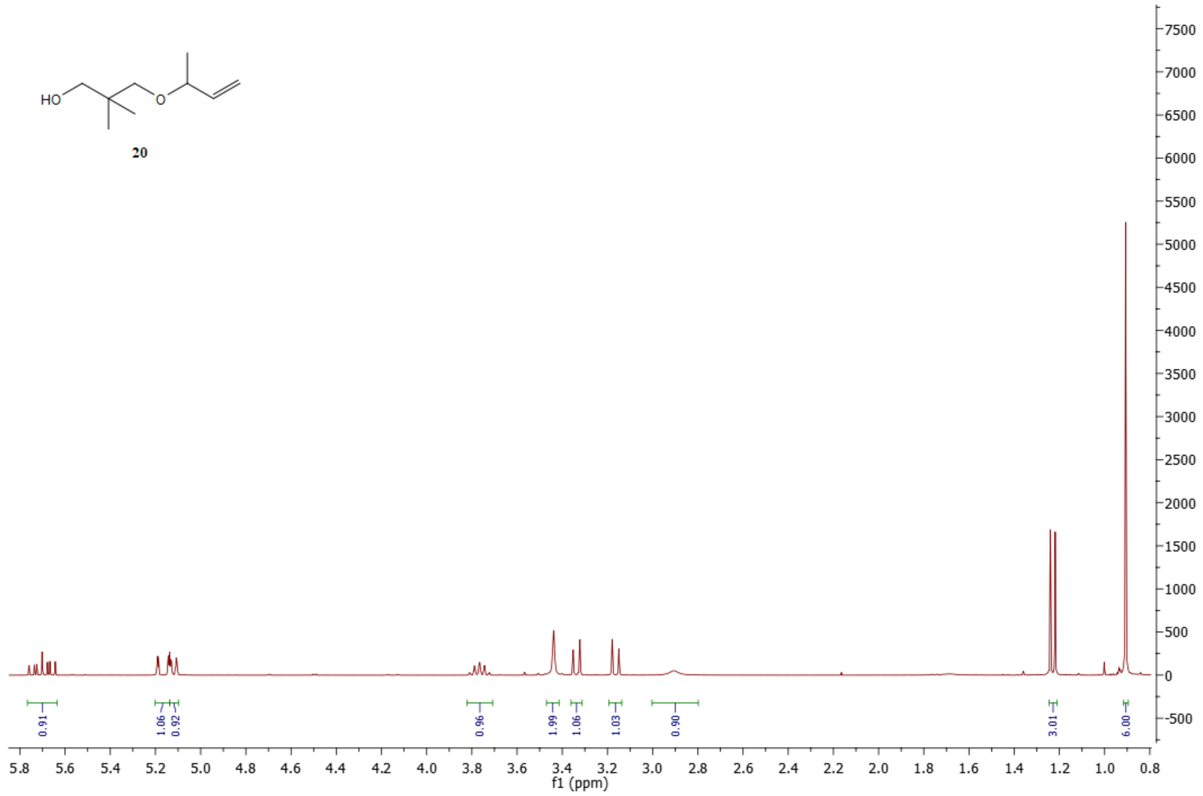
HO
O
20
f1 (ppm)

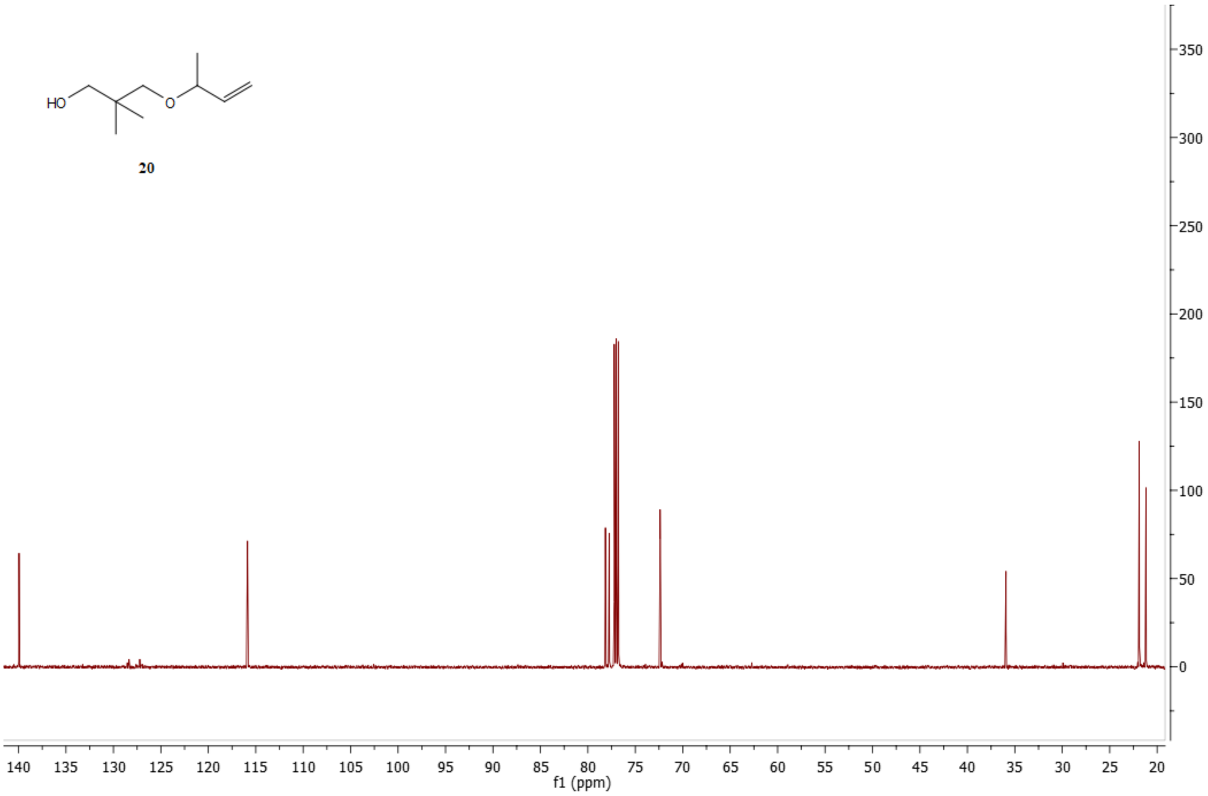
HO
O
20
f1 (ppm)

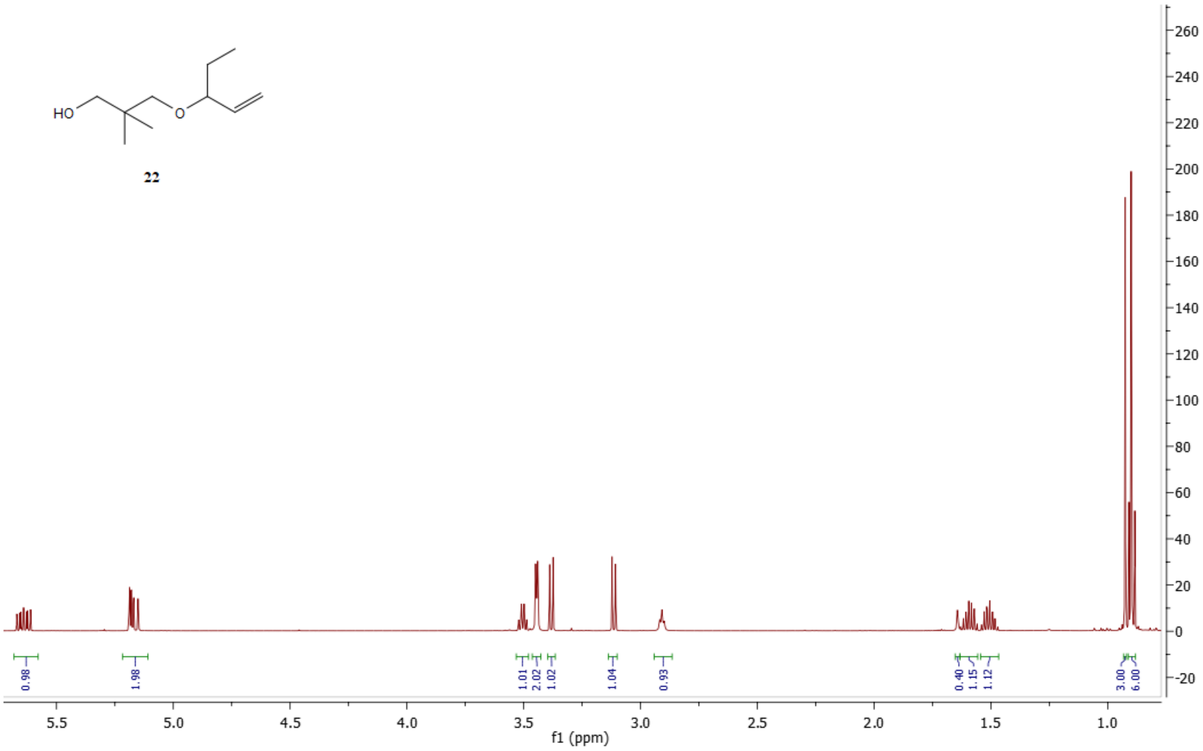
HO
O
22
0.98
1.98
1.01
2.02
1.02
1.04
0.93
0.40
1.15
1.12
3.00
6.00
f1 (ppm)

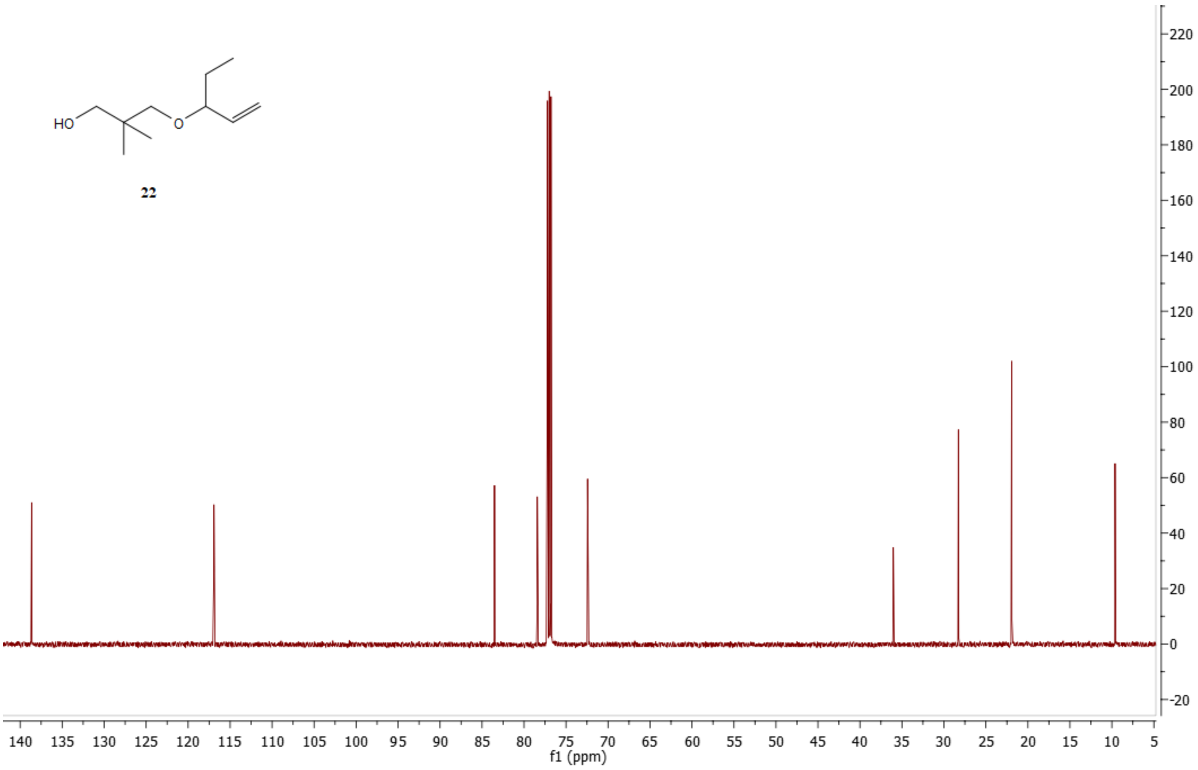
HO
O
22
f1 (ppm)

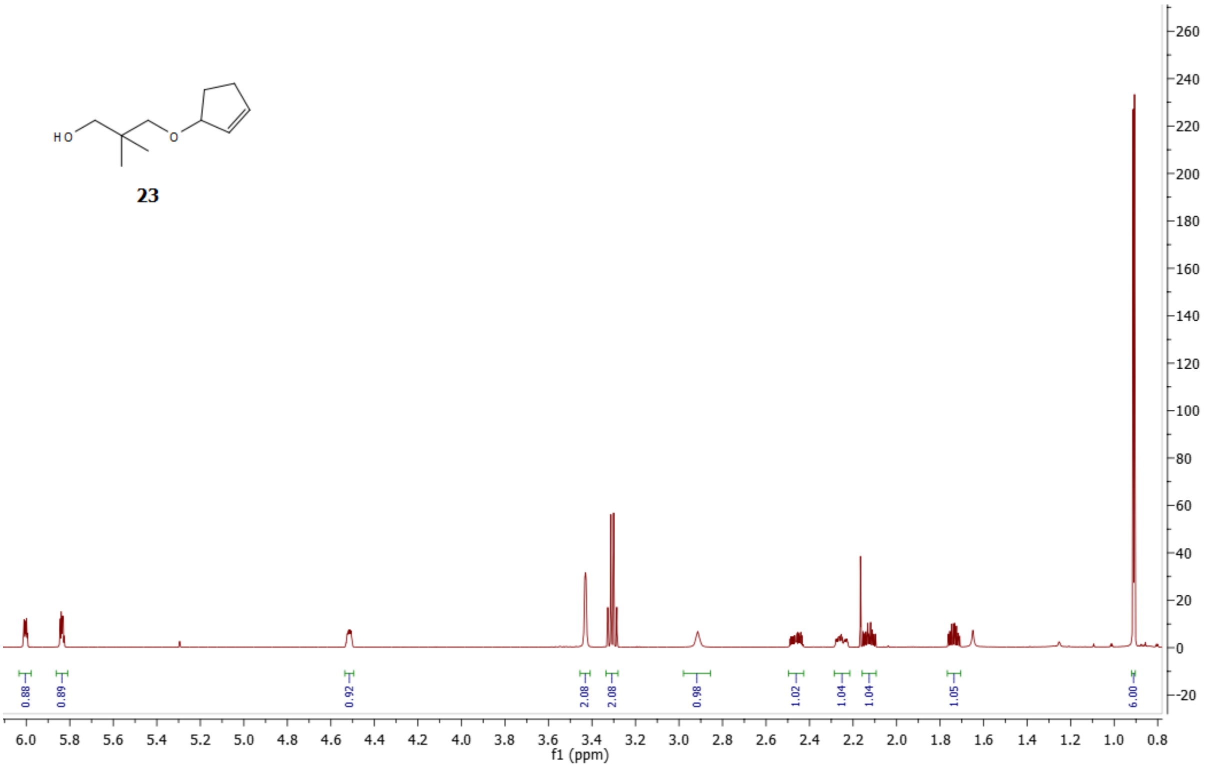
HO
O
23
0.88
0.89
0.92
2.08
2.08
0.98
1.02
1.04
1.04
1.05
6.00
6.0 5.8 5.6 5.4 5.2 5.0 4.8 4.6 4.4 4.2 4.0 3.8 3.6 3.4 3.2 3.0 2.8 2.6 2.4 2.2 2.0 1.8 1.6 1.4 1.2 1.0 0.8
f1 (ppm)
260 240 220 200 180 160 140 120 100 80 60 40 20 0 -20

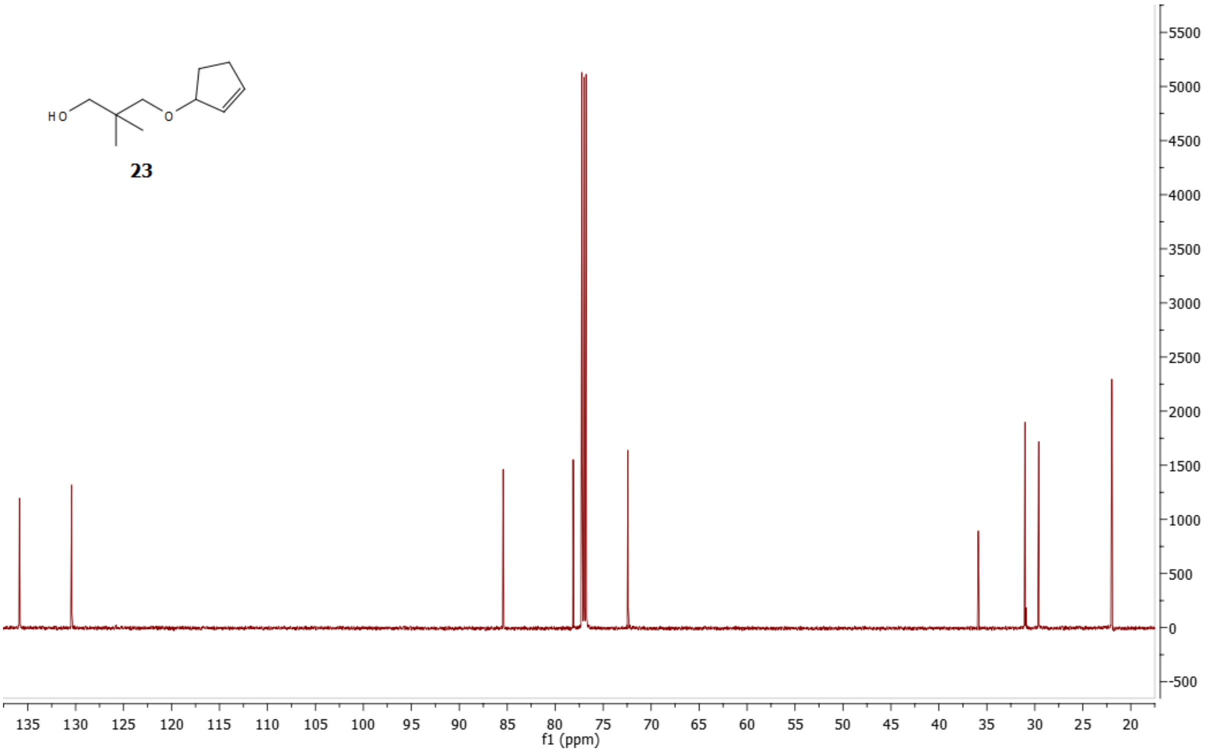
HO
O
23
135 130 125 120 115 110 105 100 95 90 85 80 75 70 65 60 55 50 45 40 35 30 25 20
f1 (ppm)
5500 5000 4500 4000 3500 3000 2500 2000 1500 1000 500 0 -500

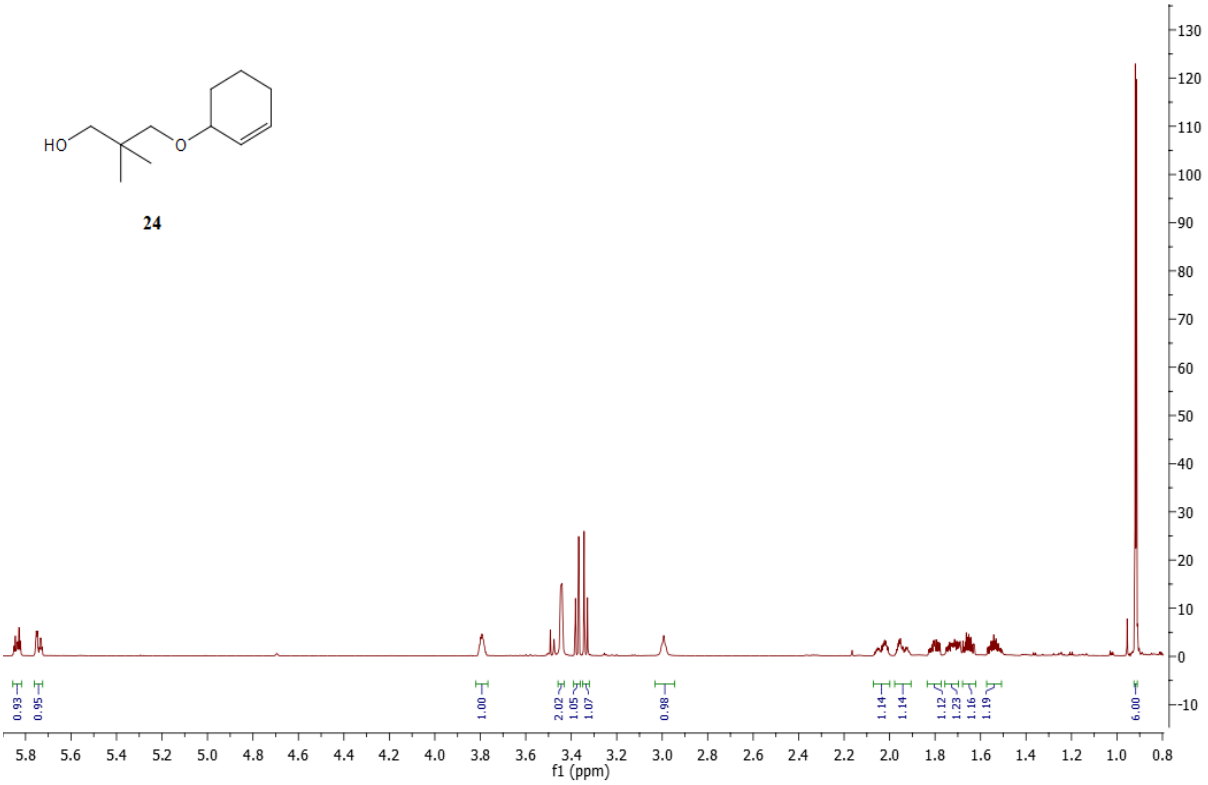

24

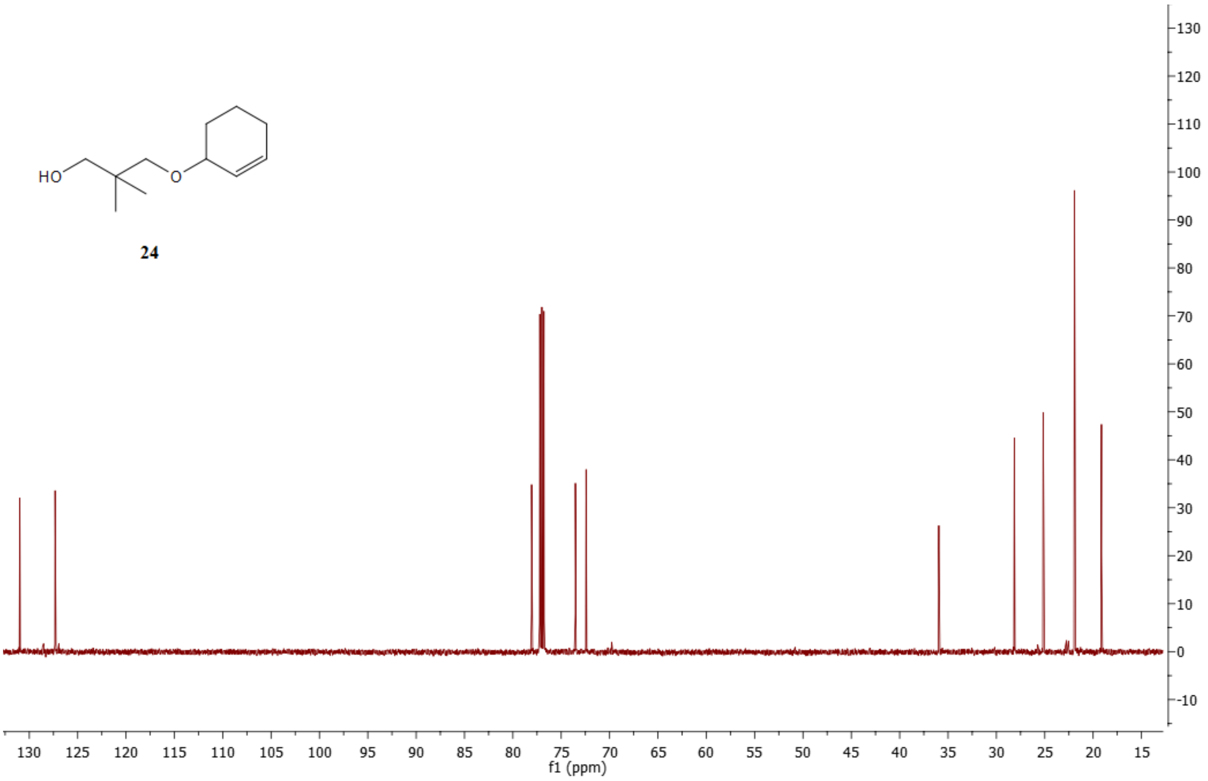

24

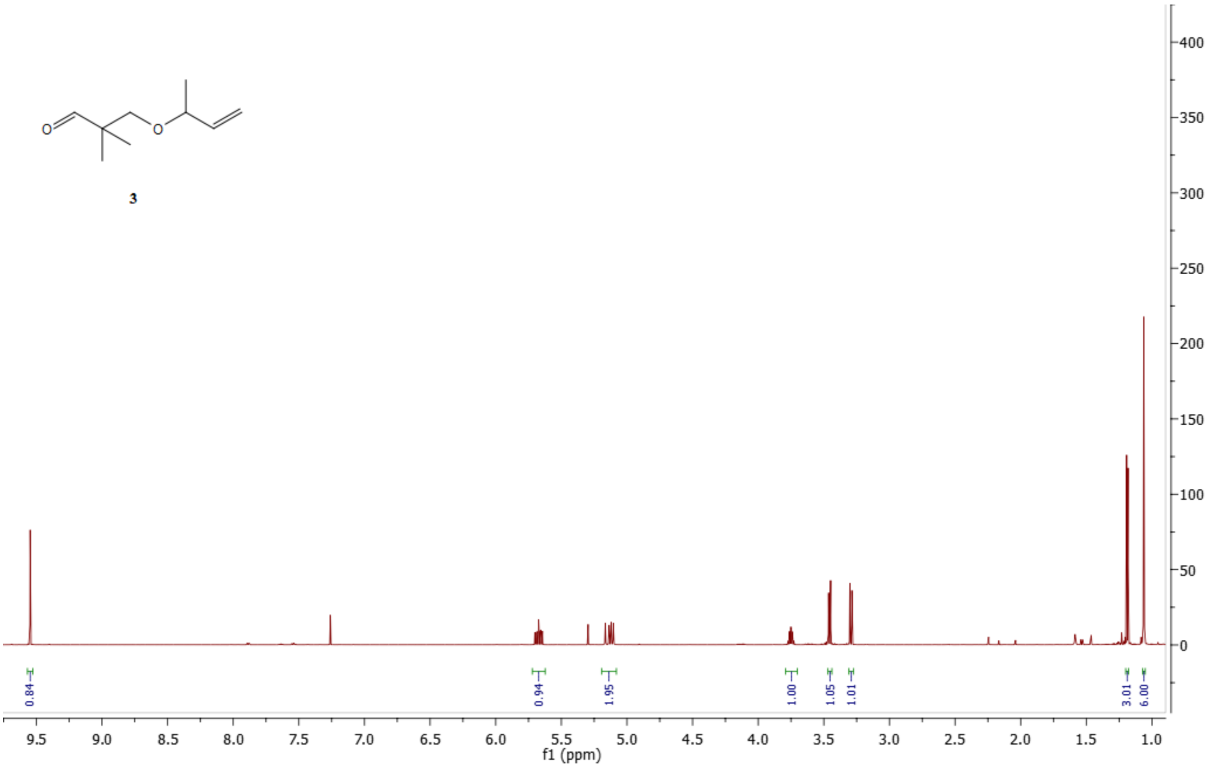
3
f1 (ppm)

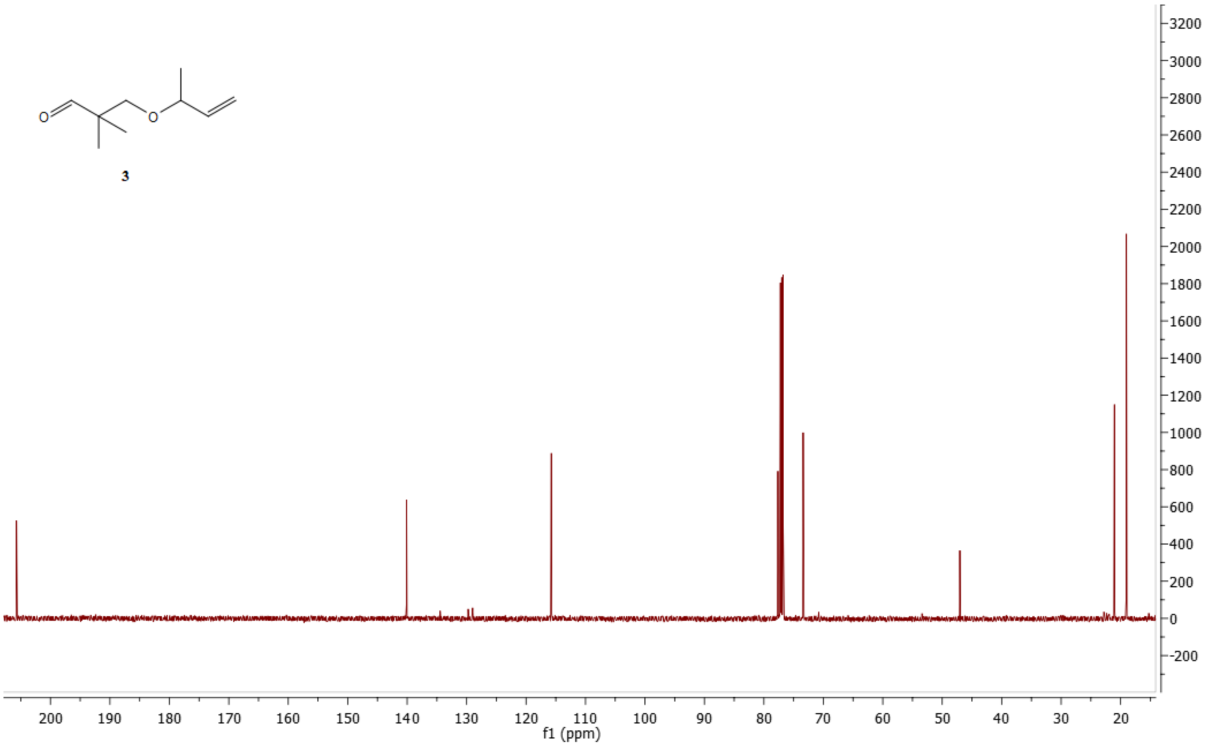
3
f1 (ppm)

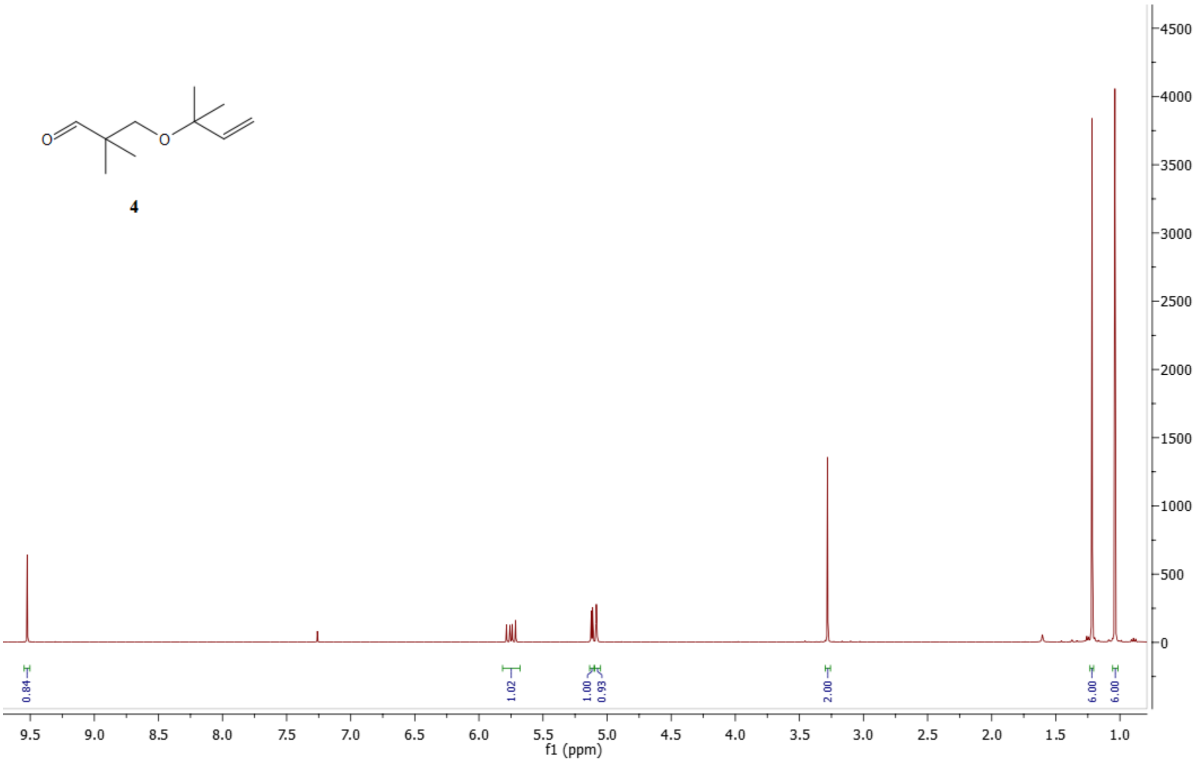

O
O
4
0.84
1.02
1.00
0.93
2.00
6.00
6.00
f1 (ppm)

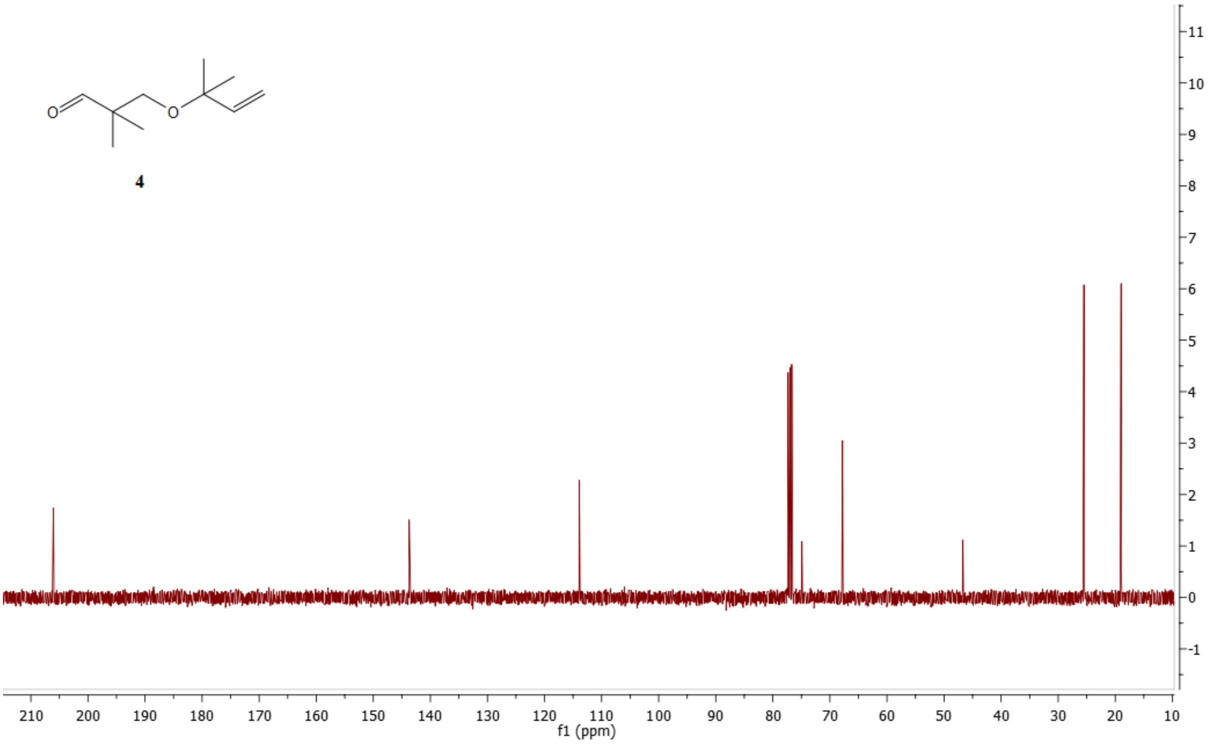

O
O
4
f1 (ppm)

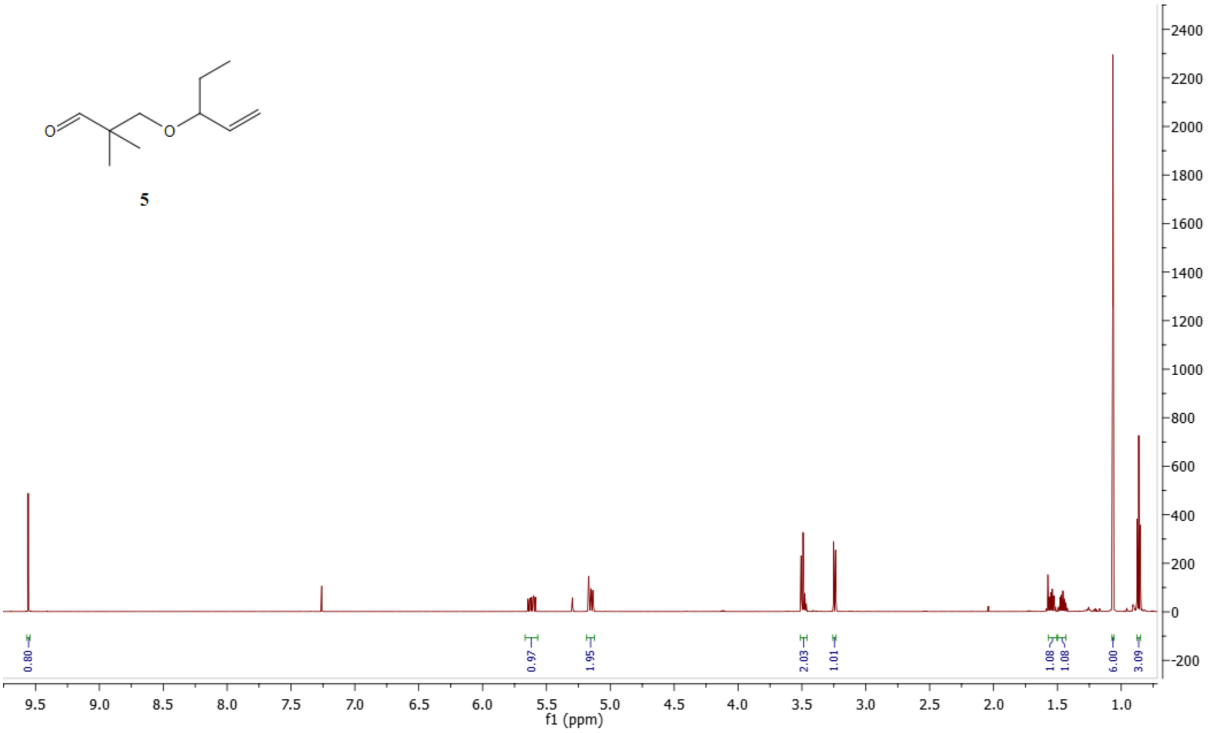

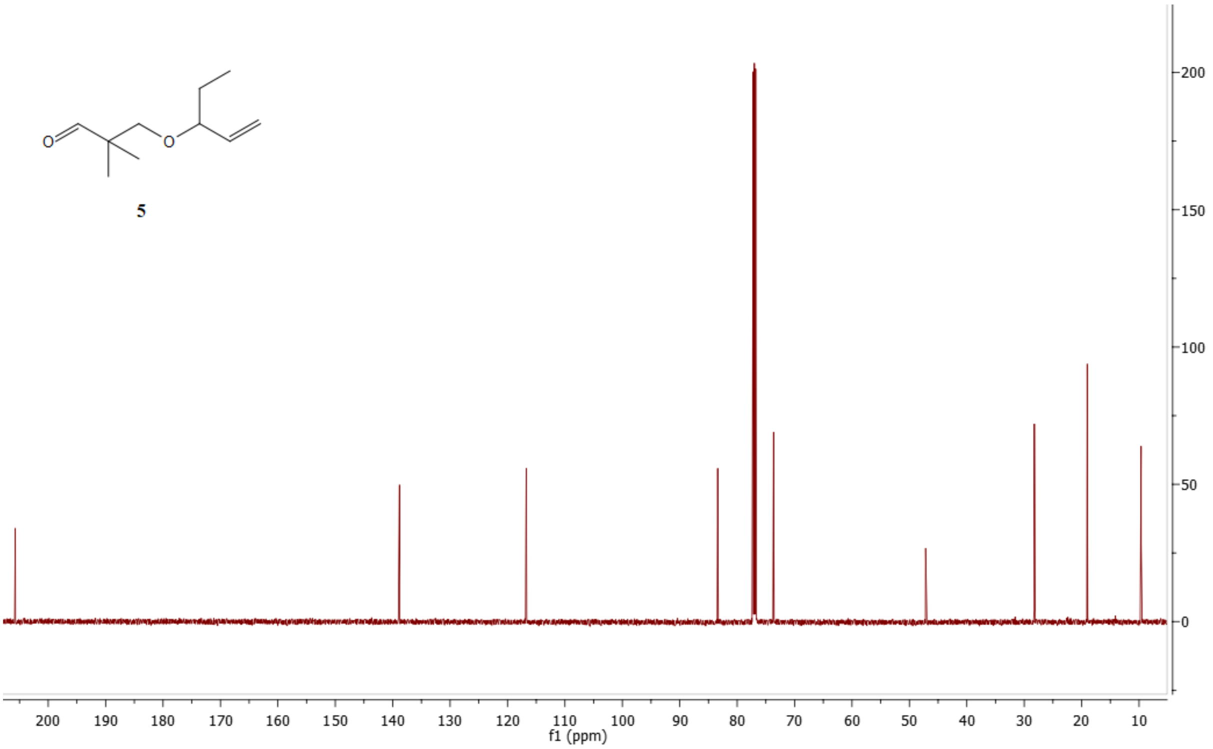

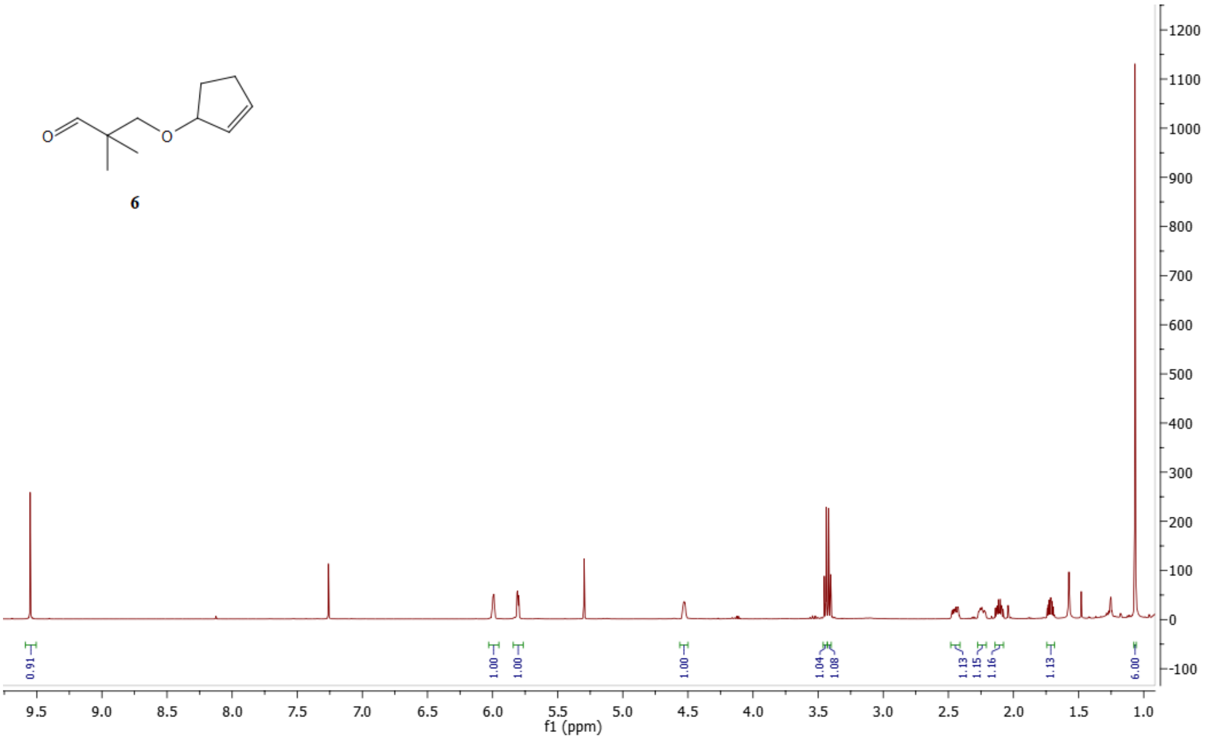
6
0.91
1.00
1.00
1.00
1.04
1.08
1.13
1.15
1.16
1.13
6.00
f1 (ppm)

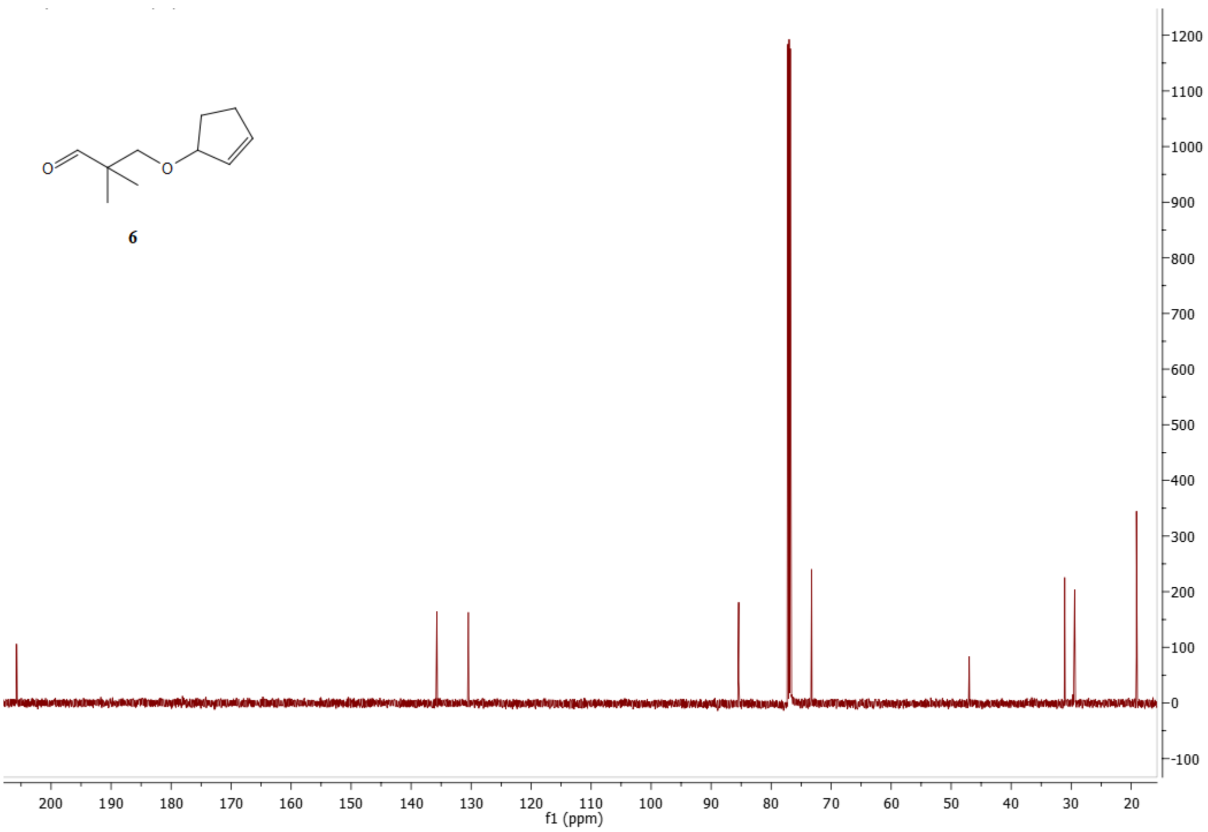
6
f1 (ppm)

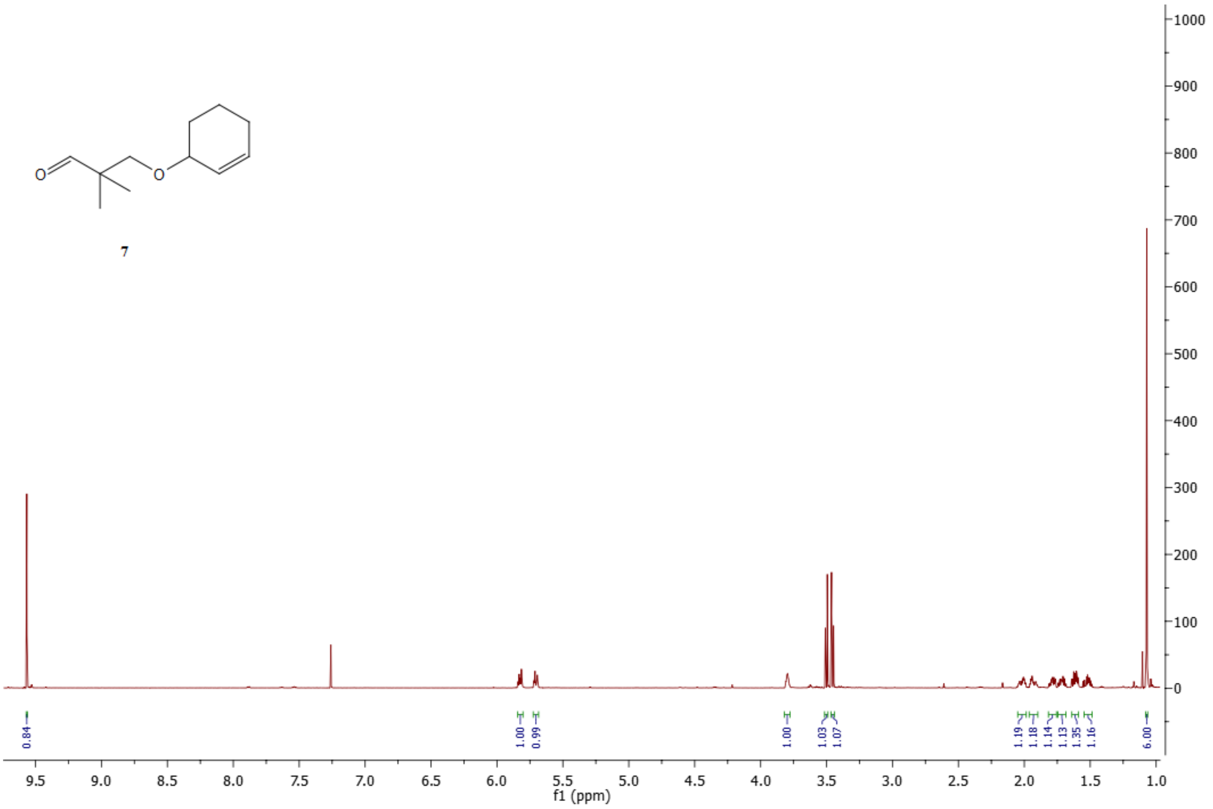

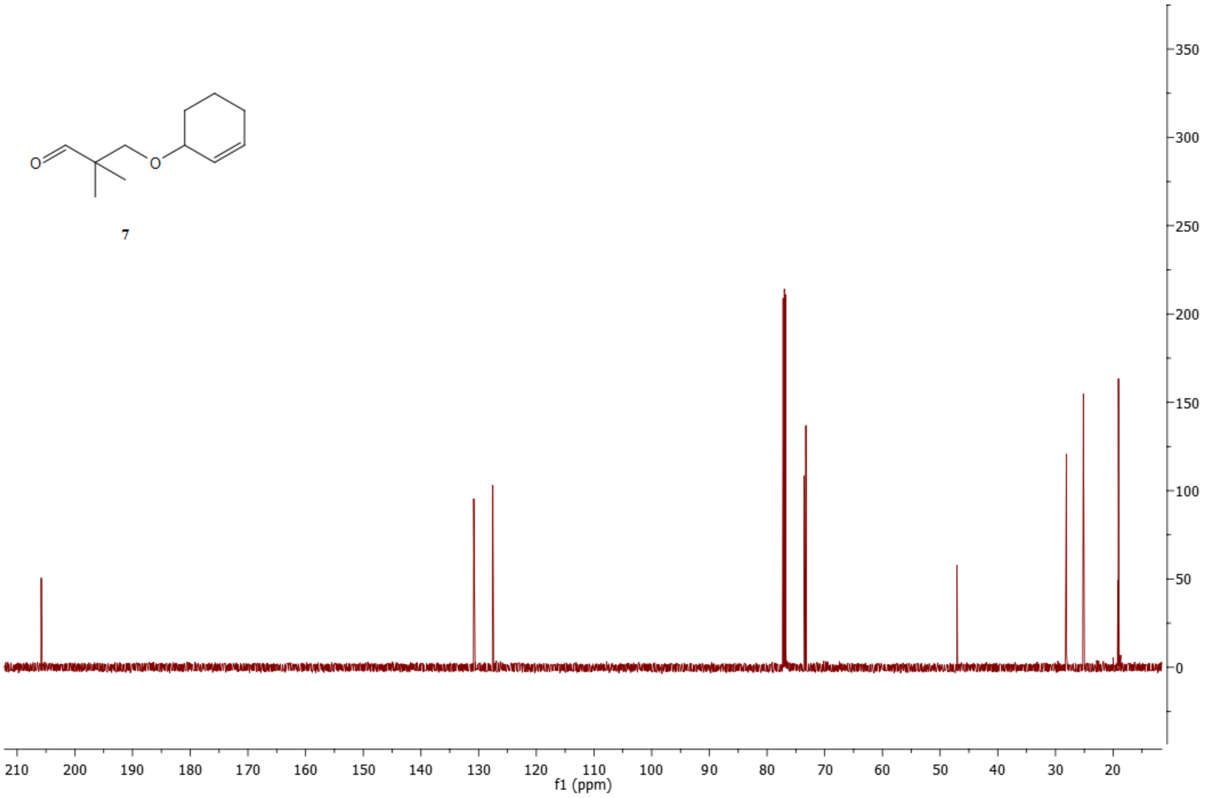

Figure S1: Copies of NMR spectra of isolated compounds
